# The association of psychological stress with metabolic syndrome and its components: cross-sectional and bidirectional two-sample Mendelian randomization analyses

**DOI:** 10.3389/fendo.2023.1212647

**Published:** 2023-12-08

**Authors:** Cancan Li, Tianqi Tao, Yanyan Tang, Huimin Lu, Hongfeng Zhang, Huixin Li, Xiuhua Liu, Weiping Guan, Yixuan Niu

**Affiliations:** ^1^ Beijing Key Laboratory of Clinical Epidemiology, School of Public Health, Capital Medical University, Beijing, China; ^2^ Department of Geriatrics, The Second Medical Center and National Clinical Research Center for Geriatric Diseases, Chinese PLA General Hospital, Beijing, China

**Keywords:** metabolic syndrome, psychological stress, hypertension, risk factor, cross-sectional study, Mendelian randomization analysis

## Abstract

**Background:**

Metabolic syndrome (MetS) is a group of co-occurring conditions that increase the risk of cardiovascular disease, which include the conditions of hypertension, overweight or obesity, hyperglycemia, and dyslipidemia. Psychological stress is gradually being taken seriously, stemming from the imbalance between environmental demands and individual perceptions. However, the potential causal relationship between psychological stress and MetS remains unclear.

**Method:**

We conducted cross-sectional and bidirectional Mendelian randomization (MR) analyses to clarify the potential causal relationship of psychological stress with MetS and its components. Multivariable logistic regression models were used to adjust for potential confounders in the cross-sectional study of the Chinese population, including 4,933 individuals (70.1% men; mean age, 46.13 ± 8.25). Stratified analyses of sexual characteristics were also performed. Bidirectional MR analyses were further carried out to verify causality based on summary-level genome-wide association studies in the European population, using the main analysis of the inverse variance-weighted method.

**Results:**

We found that higher psychological stress levels were cross-sectionally associated with an increased risk of hypertension in men (odds ratio (OR), 1.341; 95% confidence interval (CI), 1.023–1.758; p = 0.034); moreover, higher levels of hypertension were cross-sectionally associated with an increased risk of psychological stress in men and the total population (men: OR, 1.545 (95% CI, 1.113–2.145); p = 0.009; total population: OR, 1.327 (95% CI, 1.025–1.718); p = 0.032). Genetically predicted hypertension was causally associated with a higher risk of psychological stress in the inverse-variance weighted MR model (OR, 2.386 (95% CI, 1.209–4.710); *p* = 0.012). However, there was no association between psychological stress and MetS or the other three risk factors (overweight or obesity, hyperglycemia, and dyslipidemia) in cross-sectional and MR analyses.

**Conclusion:**

Although we did not observe an association between psychological stress and MetS, we found associations between psychological stress and hypertension both in cross-sectional and MR studies, which may have implications for targeting hypertension-related factors in interventions to improve mental and metabolic health. Further study is needed to confirm our findings.

## Introduction

Metabolic syndrome (MetS), also known as syndrome X or insulin resistance, is a cluster of co-occurring conditions, including hypertension, elevated fasting glucose, elevated triglycerides (TG), lowered high-density lipoprotein cholesterol (HDL-C), and abdominal obesity (1). Individuals with MetS are more susceptible to developing cardiovascular disease (CVD), type 2 diabetes mellitus, and cancers and have a higher risk of death (1, 2). MetS and MetS-related conditions are becoming major public health burdens worldwide. It is reported that over a quarter of the entire world population (about a billion people) has MetS, including one-third of the Chinese population (3, 4). Early recognition and intervention are important to prevent the development of MetS and its progression to chronic diseases, such as CVD (1, 3).

Psychological stress is a major public health challenge that can induce a range of physiological responses involving the neurological, endocrine, and immune systems (5, 6). Because both psychological stress and MetS are risk factors for CVD, their association has become a widespread concern in recent years (1, 6). Epidemiological studies suggested that psychological stress may predict the risk of MetS, hypertension, and obesity (7, 8). This could be attributed to the chronic nature of psychological stress, which can induce long-term alterations in emotional, physiological, and behavioral responses, subsequently influencing susceptibility to diseases such as MetS (9). In the context of existing Chinese studies, two focused on occupational stress (10, 11), while one focused on psychological stress with a relatively small sample size of 345 participants (7). This underscores the necessity of investigating the association between psychological stress and MetS in more extensive and representative Chinese populations. Nonetheless, some data from cross-sectional and cohort studies indicated that psychological factors, such as psychological stress, were outcomes of MetS rather than risk factors (12), while other studies reported no significant associations (13, 14). The aforementioned inconsistent results emphasize the need to investigate the causal relationship between psychological stress and MetS and its components. Such inquiry could provide a scientific foundation for developing targeted prevention policies aimed at mitigating psychological stress, MetS, and associated risk factors.

Mendelian randomization (MR) is a novel approach used to estimate the causal relationship between psychological stress and MetS using genetic variants robustly related to exposure as instrumental variables (IVs), which could overcome the limitations of observational research (15, 16). Due to the random allocation of genotypes from parents to offspring, the relationship between genetic variants and outcomes remains unaffected by common confounding factors, making a causal sequence plausible (15). Accordingly, in this current study, we aim to investigate the association of psychological stress with MetS and its components in general Chinese populations and to assess the causality using a bidirectional two-sample MR technique.

## Method

### Study design and population

This cross-sectional study was used to examine the association of psychological stress with MetS and its components, which included 4933 patients from the Chinese People’s Liberation Army General Hospital (Beijing, China) between July 2017 and June 2019. We included individuals aged 18 years and older who provided signed informed consent, had no missing data on standardized questionnaires or clinical characteristics, and were not enrolled in a clinical trial. Participants were excluded from the study if they failed to meet the inclusion criteria or had undergone surgery for cancer or other severe illnesses.

### Sample size estimation

Based on one published cross-sectional study in Asia (17), the psychological stress risk (23%) between the MetS and non-MetS groups was 24% and 22%, respectively. At 80% power (two-sided significance level of 0.05), using the sample size estimation formula for an independent sample comparison, the total sample size was estimated as:


(1) $$n=4\times {\left[\frac{({\text{Z}}_{\frac{\text{α}}{2}}+{\text{Z}}_{\text{β}})\text{σ}}{\text{δ}}\right]}^{2}=4\times {\left[\frac{(1.96+0.84)\times 23}{2}\right]}^{2}\approx 4,147$$

Consequently, the required sample size would be estimated to be 4,147. The sample size (4,933) of this current cross-sectional study meets the criteria of 4,147.

### Ethical consideration

This study conforms to the principles of the Declaration of Helsinki and relevant ethical guidelines. Approval for this study was granted by the Medical Ethics Committee of the Chinese People’s Liberation Army General Hospital (S2019-131-01).

### Data collection of demographic data and blood samples

Participants’ demographic data, including age, sex, educational attainment, marital status, smoking, alcohol consumption, physical activity, family history of diabetes, family history of hypertension, family history of CVD, and family history of stroke, was collected through face-to-face interviews with trained nurses conducting the interviews. Physical inactivity was defined as less than 2 h of physical activity per week (18). In addition, participants’ height (with a standiometer while wearing socks), body weight (with a digital weighing scale clothed in a light examination gown), waist circumference (with a measuring tape positioned at the midpoint between the lowest rib and iliac crest), and hip circumference (with a measuring tape) were measured by trained nurses. Body mass index (BMI) was calculated as weight (kg) divided by the square of height (m^2^), and the waist-to-hip ratio was calculated as waist circumference divided by hip circumference. The participants were seated for at least 5 min before two blood pressure measurements were taken by trained nurses using an automated sphygmomanometer, and the average of the two measurements was recorded.

Blood samples were collected from the antecubital vein after overnight fasting. These samples were processed, transported to the Clinical Laboratory Department of the Chinese People’s Liberation Army General Hospital, and analyzed within 24 h. Fasting blood glucose (FBG), TG, total cholesterol (TC), low-density lipoprotein cholesterol (LDL-C), and HDL-C levels were determined using a Roche C8000 automatic biochemical analyzer (Roche, Mannheim, Germany). C-reactive protein (CRP) was measured using an immunoturbidimetric assay (Siemens Healthcare Diagnostics, Germany).

### Measurement of psychological stress

The Chinese version of the Perceived Stress Scale (CPSS) was used to reflect psychological stress levels. The CPSS comprises seven positive and seven negative items rated on a 5-point Likert scale: 0 = never, 1 = rarely, 2 = sometimes, 3 = often, and 4 = always (19, 20). The total CPSS score ranges from 0 to 56, with higher scores indicating greater psychological stress; a score< 29 was defined as participants with no or low psychological stress, and a score ≥ 29 was defined as participants with moderate or high psychological stress (19, 20). The CPSS was verified in a smoking population and showed good reliability (Cronbach’s alpha = 0.85), structural validity, and co-validity (20).

### Measurement of depression and anxiety symptoms

Depressive- and anxiety-related symptoms were measured using the Chinese version of the Zung Self-Rating Depression Scale (SDS) and the Zung Self-Rating Anxiety Scale (SAS) (21, 22). Both the SDS and SAS questionnaires are composed of 20 items (10 positive and 10 negative items) scored on a 4-point scale (1 = never or rarely; 2 = sometimes; 3 = frequently; and 4 = most of the time), with higher scores representing higher depression or anxiety symptoms. The index score (range, 25–100) was equal to the raw score (range, 20–80) × 1.25, and an index score ≥ 50 was defined as participants with depression or anxiety symptoms; otherwise, they were classified as not having depression or anxiety symptoms according to the Chinese norm (21–24). Furthermore, the Chinese version of the SDS and SAS questionnaires were shown to have good reliability (Cronbach’s alpha = 0.796; Cronbach’s alpha = 0.850) and validity in the Chinese population (23, 24).

### Measurement of sleep quality

Sleep quality was assessed using the Pittsburgh Sleep Quality Index (PSQI). The PSQI consists of 19 items under seven components (subjective sleep quality, sleep latency, sleep duration, habitual sleep, efficiency, sleep disturbances, use of sleep medication, and daytime dysfunction) rated on a 4-point scale (0 = never to 3 = often). The total score on the PSQI scale ranges from 0 to 21, with a score of > 5 indicating poor sleep quality (25). The Chinese version of the PSQI has been verified in a Chinese group and has shown good reliability (Cronbach’s alpha = 0.850) and validity (26).

### Definition of MetS and its risk components

In this study, MetS was defined according to the Chinese Diabetes Society (CDS) criteria as having at least three of the following metabolic abnormalities: (1) overweight or obesity: BMI ≥ 25 kg/m^2^; (2) hypertension: systolic blood pressure (SBP) ≥ 140 mmHg, diastolic blood pressure (DBP) ≥ 90 mmHg, and (or) being treated for hypertension; (3) hyperglycemia: FBG ≥ 6.1 mmol/L, 2-h oral glucose tolerance test ≥ 7.8 mmol/L, and (or) being drug treated for type 2 diabetes; and (4) dyslipidemia: TG ≥ 1.7 mmol/L and (or) HDL-C< 0.9 mmol/L in men,< 1.0 mmol/L in women (27). The CDS has been validated in the Chinese population, showing good validity (specificity = 0.989) and reliability (28).

### Statistical analyses

The Kolmogorov–Smirnov test was performed for continuous data. Continuous data of normal distribution are represented as mean ± standard deviation (SD) ( $\bar{x}\pm s$ ), and the analysis was performed using the two independent samples *t*-test (Student’s *t*-test). Non-normally distributed continuous data were represented as median and interquartile range (IQR), and the analysis was performed using the Mann–Whitney *U* test. The chi-square test (*χ* ^2^-test) was performed to analyze categorical variables, which were expressed as frequencies, percentages, or ratios (%). The least absolute shrinkage and selection operator (Lasso) algorithm was used to screen potential confounding factors that were significantly associated with psychological stress, MetS, and its components, thus avoiding overfitting and effectively controlling the model’s complexity. Significant potential confounding factors selected with Lasso were then introduced into multivariate logistic regression analyses. SPSS (version 25, IBM) statistical software was used for statistical analysis of the data, and a two-tailed *p*-value below 0.05 was considered statistically significant.

### MR analysis

A bidirectional two-sample MR analysis was performed to evaluate the causality between psychological stress and MetS and its components (i.e., hypertension, BMI, TG, HDL-C, and FBG) to validate the cross-sectional results. MR depends on three premises: (1) genetic variation as an instrumental variable (IV) is significantly associated with exposure, (2) IVs are not related to any confounders of the exposure–outcome association, and (3) IVs can affect the outcome only via exposure (**Supplementary Figure S1**). To avoid bias due to participant overlap, this MR study relied on the largest available genome-wide association studies (GWASs) on different international consortia for exposure and outcomes. For instance, we obtained summary GWAS data associated with MetS from the most comprehensive GWAS in the UK Biobank, which included 291,107 individuals (59,677 cases and 231,430 controls) (29). Summary-level data on psychological stress were collected from the FinnGen Biobank (ID: finn-b-F5_NEUROTIC), which included 218,792 individuals (20,682 cases and 198,110 controls) (https://gwas.mrcieu.ac.uk/). The sources of GWAS data on hypertension (30), BMI, FBG (31), HDL-C, and TG (32) are shown in **Supplementary Table S1**.

The inverse variance-weighted (IVW) method, which assumes that all genetic variants are valid IVs (with no heterogeneity or horizontal pleiotropy), was used as the primary approach for evaluating potential causality (33). Thereafter, five alternative analyses (MR-Egger regression method, weighted median estimator (WME), MR pleiotropy residual sum and outlier (MR-PRESSO) weighted mode, and simple mode) were performed to assess the causal effects. Of these, the WME was regarded as a valid estimation when there was heterogeneity in the genetic variants without horizontal pleiotropy (34). MR-Egger regression was used as the main evaluation when there was heterogeneity and pleiotropy, and its intercept was used to test horizontal pleiotropy (35). Meanwhile, the MR-PRESSO global test was conducted to analyze the directional horizontal pleiotropy and identify outliers (36). For the selection of IVs, we chose single nucleotide polymorphisms (SNPs) of psychological stress that reached the genome-wide significance threshold (*p<* 1×10^−5^), MetS, and its components at *p<* 5×10^−8^. Significant SNPs at linkage disequilibrium (LD) (*r* ^2^ threshold< 0.001 within a 10-Mb window) were excluded to minimize the effect of strong LD on the results. In addition, we illustrated the magnitude of heterogeneity across all IVs using Cochran’s *Q* statistic and a funnel plot (37). Furthermore, the leave-one-out method was used for the sensitive analysis (15). The *R* ^2^ (Eq. 1: $R^{2}\;=\;2\times \text{eaf}\times \left(1-\;\text{eaf}\right)\times {\text{Beta}}^{2}$ ] and F statistics (Eq. 2: $\text{F statistic }=\frac{R^{2}\times (N-2)}{\left(1-R^{2}\right)}$ ) of each SNP were used to verify the strength of exposure, with an F statistic of > 10 indicating a lower risk of IV bias. We then summed them up to assess the *R* ^2^ and *F* statistics (38). Power calculations were performed using the mRnd software (https://cnsgenomics.com/shiny/mRnd/) (39). All data analyses were conducted using the “TwoSampleMR” and “MR-PRESSO” packages in R version 4.2.2 (R Foundation for Statistical Computing, Vienna, Austria). Statistical significance was set at a two-tailed *p*-value< 0.05.

## Results

### Cross-sectional study

#### Participants’ characteristics

**Figure 1:**
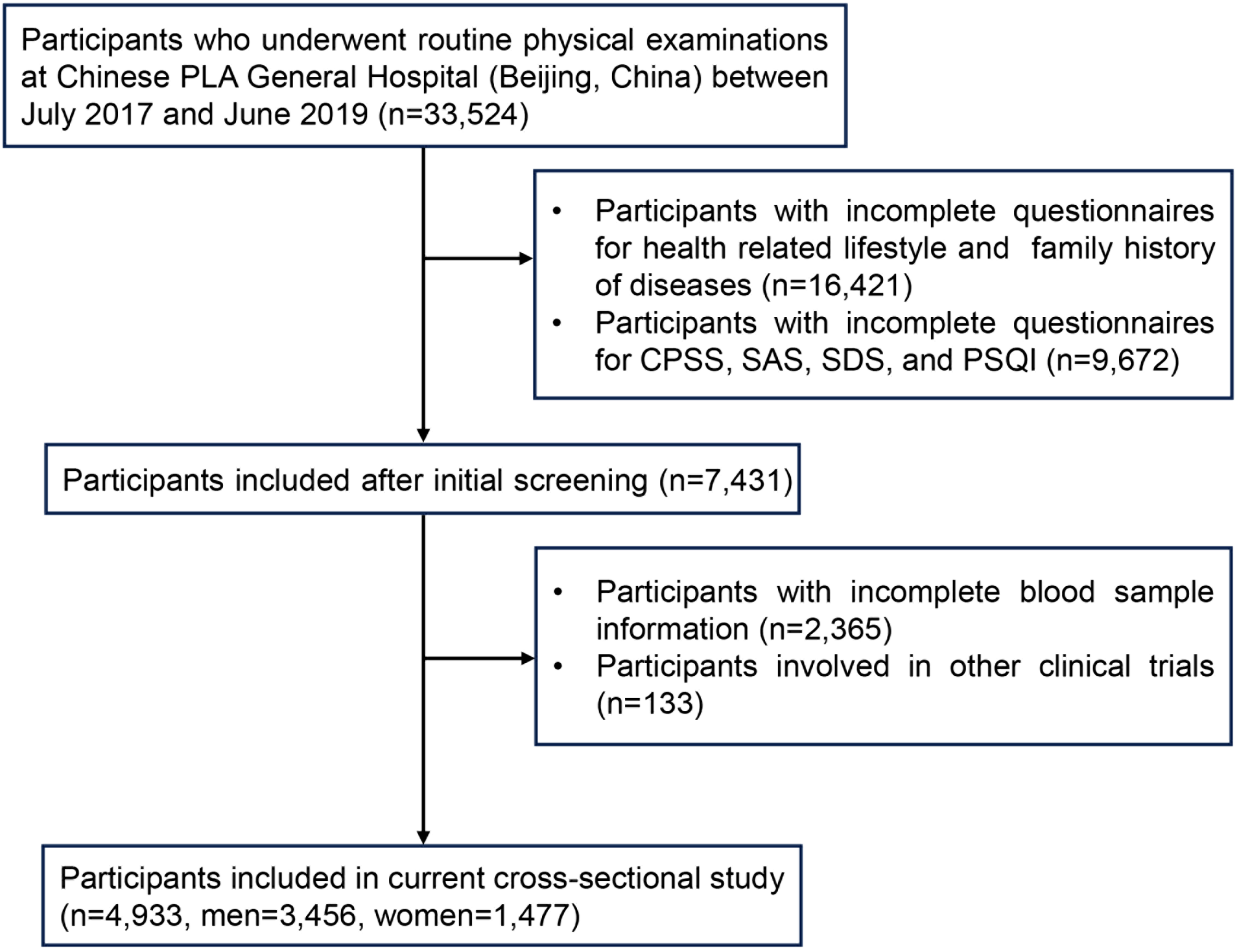
Flow chart for the selection of participants in the current cross-sectional study. Chinese PLA General Hospital, Chinese People’s Liberation Army General Hospital; CPSS, Chinese Perceived Stress Scale; SAS, Self-Rating Anxiety Scale; SDS, Self-Rating Depression Scale; PSQI, Pittsburgh Sleep Quality Index.

After excluding 28,591 individuals due to incomplete questionnaire results, incomplete blood sample information, or falling under the exclusion criteria, the data of 4,933 participants (70.1% men; mean age, 46.13 ± 8.25) were ultimately used for final analysis (**Figure 1**). Most participants had completed high school (87.4%) and were nonsmokers (70.7%). Almost all participants were married (93.2%). Health-related information revealed that the percentage of participants who reported a family history of diabetes, a family history of CVD, a family history of hypertension, and a family history of stroke were 25.0%, 22.6%, 48.3%, and 10.4%, respectively. A total of 1,489 participants (30.2%) had MetS, and 543 participants (11.0%) experienced psychological stress. The characteristics of all participants are shown in **Table 1**.

**Table 1:** Characteristics of study population with and without MetS, shown by sex.

Variables	Total (n = 4933)			Men (n = 3456)			Women (n = 1477)		
	MetS (1489, 30.2%)	Non-MetS (3444, 69.8%)	*t*/*z*/*χ^2^* (*P*)	MetS (1345, 38.9%)	Non-MetS (2111, 61.1%)	*t*/*z*/*χ^2^* (*P*)	MetS (144, 9.7%)	Non-MetS (1333, 90.3%)	*t*/*z*/*χ^2^* (*P*)
Men, n (%)	1345 (90.3)	2111 (61.3)	417.764 (<0.001)	–	–	–	–	–	–
Age (mean ± SD)	48.33 ± 7.23	45.17 ± 8.48	13.334 (<0.001)	48.07 ± 7.15	45.66 ± 8.18	9.146 (<0.001)	50.75±7.58	44.41 ± 8.90	9.365 (<0.001)
BMI, n (%)			1425.443 (<0.001)			809.457 (<0.001)			346.645 (<0.001)
< 25	136 (9.1)	2331 (67.7)		105 (7.8)	1177 (55.8)		31 (21.5)	1154 (86.6)	
≥ 25	1353 (90.9)	1113 (32.3)		1240 (92.2)	934 (44.2)		113 (78.5)	179 (13.4)	
Educational attainment, n (%)			2.622 (0.269)			3.191 (0.203)			8.365 (0.015)
Less than high school	190 (12.8)	429 (12.5)		162 (12.0)	262 (12.4)		28 (19.4)	167 (12.5)	
High school	957 (64.3)	2290 (66.5)		880 (65.4)	1427 (67.6)		77 (53.5)	863 (64.7)	
College degree or more	342 (23.0)	725 (21.1)		303 (22.5)	422 (20.0)		39 (27.1)	303 (22.7)	
Marital status, n (%)			41.737 (<0.001)			27.399 (<0.001)			3.537 (0.171)
Unmarried	20 (1.3)	181 (5.3)		16 (1.2)	92 (4.4)		4 (2.8)	89 (6.7)	
Married	1433 (96.2)	3166 (91.9)		1298 (96.5)	1976 (93.6)		135 (93.8)	1190 (89.3)	
Divorced or widowed	36 (2.4)	97 (2.8)		31 (2.3)	43 (2.0)		5 (3.5)	54 (4.1)	
Smoking, n (%)	573 (38.5)	870 (25.3)	87.801 (<0.001)	563 (41.9)	838 (39.7)	1.593 (0.207)	10 (6.9)	32 (2.4)	9.712 (0.002)
Alcohol consumption, n (%)	1037 (69.6)	1705 (49.5)	170.757 (<0.001)	1019 (75.8)	1445 (68.5)	21.458 (<0.001)	18 (12.5%)	260 (19.5%)	4.174 (0.041)
Physical activity, n (%)									
≥ 2 hours/week	696 (46.7)	1694 (49.2)	2.487 (0.115)	603 (44.8)	893 (42.3)	2.143 (0.134)	93 (64.6)	801 (60.1)	1.098 (0.295)
< 2 hours/week	793 (53.3)	1750 (50.8)		742 (55.2)	1218 (57.7)		51 (35.4)	532 (39.9)	
Waist-to-hip ratio (IQR)	0.96 (0.93, 0.99)	0.89 (0.81, 0.93)	32.820 (<0.001)	0.96 (0.93, 0.99)	0.93 (0.90, 0.95)	23.494 (<0.001)	0.84 (0.82, 0.86)	0.80 (0.75, 0.83)	13.405 (<0.001)
Family history of diabetes, n (%)	462 (31.0)	773 (22.4)	40.802 (<0.001)	427 (31.7)	466 (22.1)	40.110 (<0.001)	35 (24.3)	307 (23.0)	0.119 (0.731)
Family history of hypertension, n (%)	870 (58.4)	1514 (44.0)	87.138 (0.001)	784 (58.3)	948 (44.9)	58.852 (<0.001)	86 (59.7)	566 (42.5)	15.705 (<0.001)
Family history of CVD, n (%)	356 (23.9)	761(22.1)	1.949 (0.163)	308 (22.9)	439 (20.8)	2.146 (0.143)	48 (33.3)	322 (24.2)	5.830 (0.016)
Family history of stroke, n (%)	184 (12.4)	331 (9.6)	8.386 (0.004)	173 (12.9)	212 (10.0)	6.599 (0.010)	11 (7.6)	119 (8.9)	0.269 (0.604)
CRP (IQR)	0.12 (0.06, 0.22)	0.08 (0.05, 0.14)	10.556 (<0.001)	0.11 (0.06, 0.23)	0.09 (0.05, 0.15)	7.673 (<0.001)	0.15 (0.07, 0.26)	0.08 (0.05, 0.13)	7.024 (<0.001)
CPSS, n (%)			14.861 (<0.001)			2.776 (0.096)			1.288 (0.256)
< 29	1364 (91.6)	3026 (87.9)		1238 (92.0)	1908 (90.4)		126 (87.5)	1118 (83.9)	
≥ 29	125 (8.4)	418 (12.1)		107 (8.0)	203 (9.6)		18 (12.5)	215 (16.1)	
CPSS (IQR)	17.0 (12.0, 22.0)	18.0 (13.0, 24.0)	3.561 (<0.001)	17.0 (12.0, 22.0)	18.0 (13.0, 23.0)	1.899 (0.058)	20.0 (13.0, 25.0)	19.0 (14.0, 25.0)	0.239 (0.811)
SAS, n (%)			6.201 (0.013)			1.484 (0.223)			4.571 (0.033)
< 50	1284 (86.2)	2873 (83.4)		1182 (87.9)	1825 (86.5)		102 (70.8)	1048 (78.6)	
≥ 50	205 (13.8)	571 (16.6)		163 (12.1)	286 (13.5)		42(29.2)	285 (21.4)	
SDS, n (%)			0.009 (0.923)			2.521 (0.112)			5.644 (0.018)
< 50	1006 (67.6)	2322 (67.4)		934 (69.4)	1519 (72.0)		72 (50.0)	803 (60.2)	
≥ 50	483 (32.4)	1122 (32.6)		411 (30.6)	592 (28.0)		72 (50.0)	530 (39.8)	
PSQI, n (%)			4.171 (0.041)			0.438 (0.508)			0.084 (0.772)
≤5	553 (37.1)	1175 (34.1)		512 (38.1)	780 (36.9)		41 (28.5)	395 (29.6)	
>5	936 (62.9)	2269 (65.9)		833 (61.9)	1331 (63.1)		103 (71.5)	938 (70.4)	

MetS, metabolic syndrome; SD, standard deviation; BMI, Body Mass Index; IQR, interquartile range; CVD, cardiovascular diseases; CRP, C-reactive protein; CPSS, Chinese Perceived Stress Scale; SAS, Self-Rating Anxiety Scale; SDS, Self-Rating Depression Scale; PSQI, Pittsburgh Sleep Quality Index.

#### Descriptive data and comparison of all variables in participants with and without MetS by sex

According to the CDS criteria, the percentage of participants who reported hypertension, overweight or obesity, hyperglycemia, and dyslipidemia were 39.8%, 50.0%, 41.5%, and 44.2%, respectively. Overall, the prevalence of MetS among participants was 30.2%. Notably, MetS was present in 1,345 (38.9%) and 144 (9.7%) men and women, respectively (*p*< 0.001). There were significant differences in CPSS, SAS, and PSQI scores between participants with and without MetS in the total population (*p*< 0.001). Compared to participants without MetS, those with MetS were older (*p*< 0.001), had higher rates of smoking (*p*< 0.001) and alcohol consumption (*p*< 0.001), higher CRP values (*p*< 0.001), and family histories of diabetes, hypertension, and stroke (*p*< 0.001) in both sexes. In addition, the prevalence of participants with low educational attainment and a family history of CVD was higher in women with MetS than in those without MetS. Further information is provided in **Table 1**.

#### Descriptive data and comparison of all variables in participants with and without psychological stress by sex

As shown in **Table 2**, the prevalence of psychological stress (11.0%) in women (15.8%) was higher than that in men (9.0%) (*p*< 0.001). For MetS and its components, there were significant differences in MetS, hyperglycemia, overweight or obesity, dyslipidemia, SBP, DBP, FBG, TG, and HDL between individuals with and without psychological stress in the total population, but not in subgroup analysis by sex (*p*< 0.05). For the potential confounding factors, compared to participants without psychological stress, those with psychological stress had significant differences in age, marital status, waist-to-hip ratio, SAS, SDS, and PSQI (*p*< 0.05). Further information is shown in **Table 2**.

**Table 2:** Characteristics of study population according to the presence of psychological stress, shown by sex.

Variable	Total (4933)			Men (3456, 70.1%)			Women (1477, 29.9%)		
	Stressed (543, 11.0%)	Non-stressed (4390, 89.0%)	*t*/*z*/*χ^2^* (*P*)	Stressed (310, 9.0%)	Non-stressed (3146, 91.0%)	*t*/*z*/*χ^2^* (*P*)	Stressed (233, 15.8%)	Non-stressed (1244, 84.2%)	*t*/*z*/*χ^2^* (*P*)
Men, n (%)	310 (57.1 %)	3146 (71.7%)	48.921 (<0.001)	–	–	–	–	–	–
Age (mean ± SD)	42.74 ± 9.04	46.55 ± 8.05	9.372 (<0.001)	43.17 ± 8.70	46.93 ±7.71	7.346 (<0.001)	42.16 ± 9.46	45.56 ± 8.79	5.091 (<0.001)
BMI, n (%)			23.644 (<0.001)			4.924 (0.026)			0.824 (0.364)
< 25	325 (59.9)	2142 (48.8)		133 (42.9)	1149 (36.5)		192 (82.4)	993 (79.8)	
≥ 25	218 (40.1)	2248(51.2)		177 (57.1)	1997 (63.5)		41 (17.6)	251 (20.2)	
Educational attainment, n (%)			5.626 (0.060)			3.317 (0.190)			1.521 (0.467)
Less than high school	72 (13.3)	547 (12.5)		41 (13.2)	383 (12.2)		31 (13.3)	164 (13.2)	
High school	334 (61.5)	2913 (66.4)		193 (62.3)	2114 (67.2)		141 (60.5)	799 (64.2)	
College degree or more	137 (25.2)	930 (21.2)		76 (24.5)	649 (20.6)		61 (26.2)	281 (22.6)	
Marital status, n (%)			44.252 (<0.001))			27.529 (<0.001)			11.175 (0.004)
Unmarried	51 (9.4)	150 (3.4)		25 (8.1)	83 (2.6)		26 (11.2)	67(5.4)	
Married	477 (87.8)	4122 (93.9)		278 (89.7)	2996 (95.2)		199 (85.4)	1126 (90.5)	
Divorced or widowed	15 (2.8)	118 (2.7)		7 (2.3)	67 (2.1)		8 (3.4)	51 (4.1)	
Smoking, n (%)	167 (30.8)	1276 (29.1)	0.666 (0.414)	155 (50.0)	1246 (39.6)	12.648 (<0.001)	12 (5.2)	30 (2.4)	5.328 (0.021)
Alcohol consumption, n (%)	253 (46.6)	2489 (56.7)	19.983 (<0.001)	205 (66.1)	2259 (71.8)	4.443 (0.035)	48 (20.6)	230 (18.5)	0.573 (0.449)
Physical activity, n (%)	227 (41.8)	2163 (49.3)	10.785 (0.001)	98 (31.6)	1398 (44.4)	18.905 (<0.001)	129 (55.4)	765 (61.5)	3.087 (0.079)
Waist-to-hip ratio (IQR)	0.87 (0.80, 0.93)	0.92 (0.84, 0.96)	8.902 (<0.001)	0.93 (0.90, 0.96)	0.94 (0.92, 0.97)	4.605 (<0.001)	0.80 (0.74, 0.82)	0.81 (0.77, 0.83)	3.020 (0.003)
Family history of diabetes, n (%)	139 (25.6)	1096 (25.0)	0.103 (0.748)	79 (25.5)	814 (25.9)	0.022 (0.881)	60 (25.8)	282 (22.7)	1.048 (0.306)
Family history of hypertension, n (%)	252 (46.4)	2132 (48.6)	0.900 (0.343)	147 (47.4)	1585 (50.4)	0.990 (0.320)	105 (45.1)	547 (44.0)	0.095 (0.758)
Family history of CVD, n (%)	129 (23.8)	988 (22.5)	0.432 (0.511)	74 (23.9)	673 (21.4)	1.023 (0.312)	55 (23.6)	315 (25.3)	0.308 (0.579)
Family history of stroke, n (%)	50 (9.2)	465 (10.6)	0.990 (0.320)	30 (9.7)	355 (11.3)	0.736 (0.391)	20 (8.6)	110 (8.8)	0.016 (0.898)
CRP (IQR)	0.09 (0.05, 0.15)	0.09 (0.05, 0.16)	0.304 (0.761)	0.10 (0.05, 0.19)	0.10 (0.05, 0.18)	0.447 (0.655)	0.08 (0.05, 0.13)	0.08 (0.05, 0.15)	0.371 (0.752)
MetS, n (%)	125 (23.0)	1364 (31.1)	14.861 (<0.001)	107 (34.5)	1238 (39.4)	2.776 (0.096)	18 (7.7)	126 (10.1)	1.288 (0.256)
Hypertension, n (%)	205 (37.8)	1756 (40.0)	1.019 (0.313)	154 (49.7)	1472 (46.8)	0.945 (0.331)	51 (21.9)	284 (22.8)	0.099 (0.753)
Hyperglycemia, n (%)	193 (35.5)	1854 (42.2)	8.906 (0.003)	127 (41.0)	1421 (45.2)	2.014 (0.156)	66 (28.3)	433 (34.8)	0.685 (0.055)
Overweight or obesity, n (%)	218 (40.1)	2248 (51.2)	23.644 (<0.001)	177 (57.1)	1997 (63.5)	4.924 (0.026)	41 (17.6)	251 (20.2)	0.824 (0.364)
Dyslipidemia, n (%)	204 (37.6)	1975 (45.0)	10.787 (0.001)	162 (52.3)	1717 (54.6)	0.612 (0.434)	42 (18.0)	258 (20.7)	0.893 (0.345)
SBP (IQR)	117.0 (105.0, 132.0)	122.0 (109.0, 136.0)	4.701 (<0.001)	123.0 (110.0, 137.0)	126.0 (113.0, 138.0)	1.182 (0.237)	109.0 (98.0, 122.0)	112.0 (101.0, 128.0)	3.000 (<0.001)
DBP (IQR)	79.0 (71.0, 89.0)	81.0 (74.0, 89.0)	3.123 (<0.001)	83.0 (75.0, 92.0)	82.0 (75.0, 90.0)	0.884 (0.377)	74.0 (67.0, 82.0)	78.0 (71.0, 85.0)	3.880 (<0.001)
FBG (mmol/L, IQR)	5.09 (4.79, 5.55)	5.24 (4.88, 5.74)	3.796 (<0.001)	5.28 (4.94, 5.83)	5.34(4.96, 5.92)	1.465 (0.143)	4.96 (4.63, 5.30)	5.00 (4.71, 5.32)	1.323 (0.186)
Triglycerides (mmol/L, IQR)	1.30 (0.91, 2.05)	1.53 (1.05, 2.29)	4.230 (<0.001)	1.68 (1.20, 2.50)	1.73 (1.22, 2.53)	0.482 (0.630)	1.00 (0.75, 1.37)	1.08 (0.80, 1.52)	2.454 (0.014)
HDL (mmol/L, IQR)	1.29 (1.06, 1.53)	1.20 (1.00, 1.46)	3.717 (<0.001)	1.14 (0.95, 1.32)	1.13 (0.96, 1.33)	0.439 (0.661)	1.48 (1.25, 1.76)	1.48 (1.23, 1.74)	0.812 (0.417)
LDL (mmol/L, IQR)	3.03 (2.44, 3.66)	3.11 (2.54, 3.69)	1.325 (0.185)	3.20 (2.57, 3.75)	3.11 (2.54, 3.70)	0.896 (0.370)	2.94 (2.36, 3.60)	3.12 (2.55, 3.70)	3.097 (0.002)
CHO (mmol/L, IQR)	4.61 (3.99, 5.19)	4.70 (4.11, 5.31)	1.910 (0.056)	4.63 (4.07, 5.30)	4.69(4.09, 5.31)	0.008 (0.994)	4.64 (3.94, 5.15)	4.75 (4.15, 5.33)	3.084 (0.002)
SAS, n (%)			745.856 (<0.001)			427.120 (<0.001)			269.124 (<0.001)
< 50	239 (44.0)	3918 (89.2)		153 (49.4)	2854 (90.7)		86 (36.9)	1064 (85.5)	
≥ 50	304 (56.0)	472 (10.8)		157 (50.6)	292 (9.3)		147 (63.1)	180 (14.5)	
SDS, n (%)			735.605 (<0.001)			457.243 (<0.001)			246.306 (<0.001)
< 50	87 (16.0)	3241 (73.8)		57 (18.4)	2396 (76.2)		30 (12.9)	845 (67.9)	
≥ 50	456 (84.0)	1149 (26.2)		253 (81.6)	750 (23.8)		203 (87.1)	399 (32.1)	
PSQI, n (%)			161.350 (<0.001)			91.845 (<0.001)			60.693 (<0.001)
≤5	57 (10.5)	1671 (38.1)		38 (12.3)	1254 (39.9)		19 (8.2)	417 (33.5)	
>5	486 (89.5)	2719 (61.9)		272 (87.7)	1892 (60.1)		214 (91.8)	827 (66.5)	

MetS, metabolic syndrome; SD, standard deviation; BMI, Body Mass Index; IQR, interquartile range; CVD, cardiovascular diseases; CHO, total cholesterol; HDL-C, high-density lipoprotein cholesterol; LDL-C, low-density lipoprotein cholesterol; FBG, fasting blood-glucose; TG, Triglycerides; CRP, C-reactive protein; SBP, systolic blood pressure; DBP, diastolic blood pressure; CPSS, Chinese Perceived Stress Scale; SAS, Self-Rating Anxiety Scale; SDS, Self-Rating Depression Scale; PSQI, Pittsburgh Sleep Quality Index.

#### Associations among psychological stress and MetS and its components

**Figure 2:**
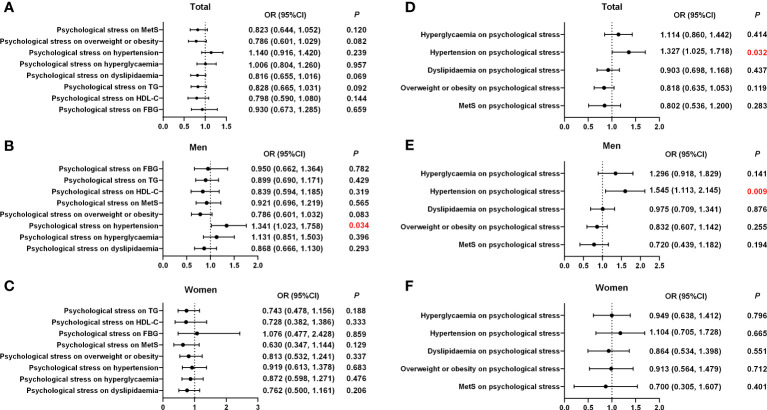
Associations of psychological stress with MetS and its components according to sex. **(A)** The effect of psychological stress on MetS and its components in the total population. **(B)** The effect of psychological stress on MetS and its components in men. **(C)** The effect of psychological stress on MetS and its components in women. **(D)** The effect of MetS and its components on psychological stress in the total population. **(E)** The effect of MetS and its components on psychological stress in men. **(F)** The effect of MetS and its components on psychological stress in women. OR, odd ratio; CI, confidence interval; MetS, metabolic syndrome.

Logistic regression models with MetS and its risk components as dependent variables were used to assess whether psychological stress was associated with MetS, overweight or obesity, hypertension, hyperglycemia, and dyslipidemia, after adjusting for potential confounding factors (age, marital status, smoking, alcohol consumption, physical activity, SAS, SDS, family history of diabetes, and family history of hypertension) selected via Lasso. The results indicated that psychological stress was linked to the risk of hypertension (odds ratio (OR), 1.341 (95% confidence interval (CI), 1.023–1.758); p = 0.034) in men (**Table 3**; **Figure 2**). In contrast, psychological stress was not associated with MetS or the three other components. Further information is provided in **Table 3**; **Figure 2**. Additionally, logistic regression models with psychological stress as dependent variables were used to assess whether MetS and its individual risk components were independent risk factors for psychological stress. These models were adjusted for age, marital status, smoking, alcohol consumption, physical activity, SAS, and SDS, which were also selected via Lasso. The results indicated that hypertension could be an independent risk factor in total participants and men (total population: OR, 1.327 (95% CI, 1.025–1.718); *p* = 0.032; men: OR, 1.545 (95% CI, 1.113–2.145); *p* = 0.009). Further information is shown in **Table 4**; **Figure 2**.

**Table 3:** Multivariate analysis of psychological stress on MetS and its risk components, shown by sex.

Variables	Total		Men		Women	
	OR (95% CI)	*p*-value	OR (95% CI)	*p*-value	OR (95% CI)	*p*-value
MetS	0.823 (0.644, 1.052)	0.120	0.921 (0.696, 1.219)	0.565	0.630 (0.347, 1.144)	0.129
Hypertension	1.140 (0.916, 1.420)	0.239	1.341 (1.023, 1.758)	0.034	0.919 (0.613, 1.378)	0.683
Overweight or obesity	0.786 (0.601, 1.029)	0.082	0.786 (0.601, 1.032)	0.083	0.813 (0.532, 1.241)	0.337
Dyslipidemia	0.816 (0.655, 1.016)	0.069	0.868 (0.666, 1.130)	0.293	0.762 (0.500, 1.161)	0.206
TG (≥ 1.7, mmol/L)	0.828 (0.665, 1.031)	0.092	0.899 (0.690, 1.171)	0.429	0.743 (0.478, 1.156)	0.188
HDL-C (< 0.9 in men,< 1.0 in women, mmol/L)	0.798 (0.590, 1.080)	0.144	0.839 (0.594, 1.185)	0.319	0.728 (0.382, 1.386)	0.333
Hyperglycemia	1.006 (0.804, 1.260)	0.957	1.131 (0.851, 1.503)	0.396	0.872 (0.598, 1.271)	0.476
FBG (≥ 6.1, mmol/L)	0.930 (0.673, 1.285)	0.659	0.950 (0.662, 1.364)	0.782	1.076 (0.477, 2.428)	0.859

All associations were tested using logistic regression, and all results of multivariate analysis were adjusted by age, marital status, smoking, alcohol consumption, physical activity, family history of diabetes, family history of hypertension, SAS, and SDS. MetS, metabolic syndrome; OR, odd ratio; CI, confidence interval; SAS, Self-Rating Anxiety Scale; SDS, Self-Rating Depression Scale.

**Table 4:** Multivariate analysis of MetS and its risk components on psychological stress, shown by sex.

Variables	Total		Men		Women	
	OR (95% CI)	*p*-value	OR (95% CI)	*p*-value	OR (95% CI)	*p*-value
MetS	0.802 (0.536, 1.200)	0.283	0.720 (0.439, 1.182)	0.194	0.700 (0.305, 1.607)	0.401
Hypertension	1.327 (1.025, 1.718)	0.032	1.545 (1.113, 2.145)	0.009	1.104 (0.705,1.728)	0.665
Hyperglycemia	1.114 (0.860, 1.442)	0.414	1.296 (0.918, 1.829)	0.141	0.949 (0.638, 1.412)	0.796
Overweight or obesity	0.818 (0.635, 1.053)	0.119	0.832 (0.607, 1.142)	0.255	0.913 (0.564, 1.479)	0.712
Dyslipidemia	0.903 (0.698, 1.168)	0.437	0.975 (0.709, 1.341)	0.876	0.864 (0.534, 1.398)	0.551

All results of multivariate analysis were adjusted by age, marital status, smoking, alcohol consumption, physical activity, SAS, and SDS. MetS, metabolic syndrome; OR, odd ratio; CI, confidence interval; SAS, Self-Rating Anxiety Scale; SDS, Self-Rating Depression Scale.

### MR analysis

#### The causal effect of psychological stress on MetS and its components

Among the 40 psychological stress-associated variants (*p*< 1 × 10^−5^, LD *r* ^2^< 0.001) (**Supplementary Table S2**), two SNPs were not available in the summary-level datasets of MetS and hypertension, 21 SNPs were unavailable for the overweight dataset, 17 SNPs were unavailable for the obesity dataset, 20 SNPs were unavailable for the BMI dataset, and 23 SNPs were unavailable for the hyperlipidemia dataset and HDL-C dataset. In addition, owing to incompatible alleles and ambiguous palindromes, we excluded two variants of MetS, hypertension, overweight, obesity, BMI, hyperlipidemia, TG, FBG, and HDL-C. Therefore, we included 36, 36, 17, 21, 18, 38, 15, 15, and 38 variants as IVs for MetS, hypertension, overweight, obesity, BMI, FBG, hyperlipidemia, HDL-C, and TG levels, respectively, in the MR analyses.

**Figure 3:**
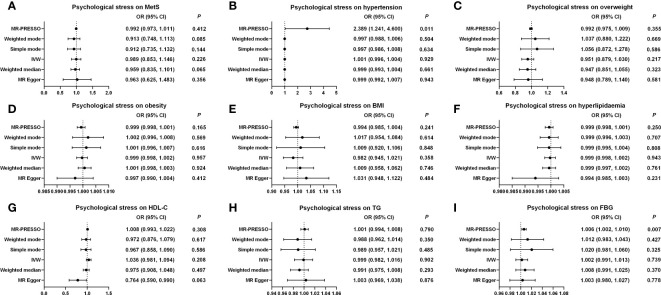
Causal estimates of genetically predicted psychological stress on MetS and its components. **(A)** Causal estimates of genetically predicted psychological stress on MetS. **(B)** Causal estimates of genetically predicted psychological stress on hypertension. **(C)** Causal estimates of genetically predicted psychological stress on overweight. **(D)** Causal estimates of genetically predicted psychological stress on obesity. **(E)** Causal estimates of genetically predicted psychological stress on BMI. **(F)** Causal estimates of genetically predicted psychological stress on hyperlipidemia. **(G)** Causal estimates of genetically predicted psychological stress on HDL-C. **(H)** Causal estimates of genetically predicted psychological stress on TG. **(I)** Causal estimates of genetically predicted psychological stress on FBG. MetS, metabolic syndrome; MR, Mendelian randomization; OR, odds ratio; CI, confidence interval; IVW, inverse-variance weighted; MR-PRESSO, MRPleiotropy Residual Sum and Outlier; BMI, body mass index; FBG, fasting blood-glucose; HDL-C, high-density lipoprotein cholesterol; TG, triglycerides.

The causations were analyzed using IVW, MR-Egger, WME, weighted mode, simple mode, and MR-PRESSO methods. As depicted in **Supplementary Table S3**; **Figure 3**; **Supplementary Figure S2**, the ORs with 95% CIs for each log-odd increment in genetically predicted causal associations between psychological stress and MetS were obtained using the IVW method (OR, 0.989 (95% CI, 0.853–1.146); *p* = 0.226). These findings were consistent with the results from the five other models. The results of the MR-Egger intercept (*p* = 0.689) and MR-PRESSO global tests (*p* = 0.151) showed no indication of potential horizontal pleiotropy. The Cochran’s *Q* value for the IVW model was *p* = 0.023, but the funnel plot considered no significant heterogeneity obtained from individual variants (**Supplementary Figure S3**). Moreover, leave-one-out analysis showed that no IVs influenced this causal inference after gradually eliminating any single SNP (**Supplementary Figure S4**). Similarly, no causal relationship was found between psychological stress and the MetS components. The results of the MR-Egger regression analyses, MR-PRESSO global tests, Cochran’s *Q* value of the IVW model, funnel plot, and leave-one-out analyses for MetS components are shown in **Supplementary Table S3**; **Figure 3**; **Supplementary Figures S2**-**S5**. Most IVs had an F statistic greater than 10, indicating that IV bias was unlikely to exist. The statistical power for MR of psychological stress on MetS and its components was higher than 75% (**Supplementary Table S4**).

#### The causal effect of MetS and its components on psychological stress

In the reverse MR analysis, after excluding the SNPs for palindromic alleles, palindromic alleles with intermediate allele frequencies, and unavailable SNPs in the summary-level dataset of psychological stress, we utilized 68, 66, 14, 13, 37, 11, 69, 31, and 94 variants for MetS, hypertension, overweight, obesity, BMI, hyperlipidemia, HDL-C, TG, and FBG as IVs (*p*< 5 × 10^−8^, LD *r* ^2^< 0.001), respectively (**Supplementary Tables S5**-**S13**).

**Figure 4:**
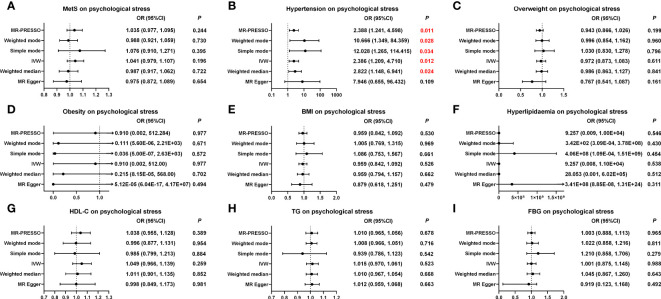
Causal estimates of genetically predicted MetS and its components on psychological stress. **(A)** Causal estimates of genetically predicted MetS on psychological stress. **(B)** Causal estimates of genetically predicted hypertension on psychological stress. **(C)** Causal estimates of genetically predicted overweight on psychological stress. **(D)** Causal estimates of genetically predicted obesity on psychological stress. **(E)** Causal estimates of genetically predicted BMI on psychological stress. **(F)** Causal estimates of genetically predicted hyperlipidemia on psychological stress. **(G)** Causal estimates of genetically predicted HDL-C on psychological stress. **(H)** Causal estimates of genetically predicted TG on psychological stress. **(I)** Causal estimates of genetically predicted FBG on psychological stress. MetS, metabolic syndrome; MR, Mendelian randomization; OR, odds ratio; CI, confidence interval; IVW, inverse-variance weighted; MR-PRESSO, Pleiotropy Residual Sum and Outlier; BMI, body mass index; FBG, fasting blood-glucose; HDL-C, high-density lipoprotein cholesterol; TG, triglycerides.

As shown in **Figure 4**, **Supplementary Table S14**; **Supplementary Figure S6**, the MR results showed that hypertension and psychological stress had a positive causal relationship in the IVW model (OR, 2.386 (95% CI, 1.209–4.710); *p* = 0.012), which was in line with the results of the WME, simple mode, weighted mode, and MR-PRESSO models. No potential horizontal pleiotropy was observed in the MR-Egger intercept test (*p* = 0.330) or the MR-PRESSO global test (*p* = 0.051). The Cochran’s *Q* value for the IVW method indicated that heterogeneity may exist (*p* = 0.021); however, the symmetry of the funnel plot showed no evidence of heterogeneity (**Supplementary Figure S7**). Furthermore, leave-one-out analysis suggested that the MR results were stable after the removal of any single SNP. Nonetheless, neither MetS nor its five other factors were causally related to psychological stress. Further information is presented in **Supplementary Tables S4**, **S14**; **Figure 4**; **Supplementary Figures S6**-**S9**.

## Discussion

MetS has been recognized as a serious health problem worldwide because of its growing prevalence (3). According to previous studies, the association between psychological stress and MetS remains unclear. In this study, we used a cross-sectional design to investigate the association of psychological stress with MetS and its risk components and used bi-directional MR analyses to explore its causal relationship. We found that psychological stress was associated with hypertension in men after controlling for potential covariates in the present cross-sectional study but not in MR analyses; conversely, hypertension was a risk factor for psychological stress in cross-sectional and MR analyses.

Psychological stress and MetS are associated with alterations in CVD; however, their relationship has not yet been fully elucidated. To reduce the limitations of observational studies, such as the disturbance of confounding effects, we used MR analysis, a scientific method, to explore the relationship between psychological stress and MetS. In the present study, we found no association between psychological stress and MetS, and similar results were obtained from the MR analyses. In line with our findings, previous cross-sectional and longitudinal studies have indicated no relationship between psychological stress and MetS, regardless of the instruments used to measure psychological pressure or the definition of MetS (40, 41). Considering salivary cortisol as an objective indicator of psychological stress, prior studies have indicated no significant difference in salivary cortisol levels between populations with and without MetS (13, 42, 43), thereby offering an interpretation of our results. Nevertheless, cross-sectional studies in Japan, Europe, and Pakistan have reported stress scores of 28, 25, and 31, respectively, observing a positive association between psychological stress and MetS (44–46). Indeed, increased psychological stress scores have been associated with an increased risk of metabolic disorders (9). Consistent with our results (mean CPSS score: 18.4), one prior cross-sectional study reporting a low stress score of 22.7 did not support the effect of stress on MetS (7).

Hypertension is a major modifiable risk factor for MetS and CVD. There is growing evidence for an association between hypertension and the progression of psychological stress (47, 48). Our cross-sectional and MR analyses revealed that hypertension may increase the risk of psychological stress. Prior research has found that hypertension causes damage to small blood vessels, contributing to neuronal damage in multiple areas, including the hippocampus, which could promote the development of psychological stress (49). One animal experiment showed that a highly activated region in the spontaneously hypertensive rat (the locus coeruleus) could awaken and regulate autonomic function and that enhanced autonomic reactivity is a true indicator of perceived stress levels (50). Therefore, it is particularly important to pay attention to the psychological stress experienced by patients with hypertension to reduce the occurrence of hypertension-related complications. Conversely, based on MR results, psychological stress may not be involved in the development of hypertension. In addition, our cross-sectional study found that psychological stress may be related to hypertension in men but found no association in women or the total population. Indeed, gender plays a role in influencing the aforementioned relationship. In the current cross-sectional survey, a higher prevalence of hypertension was observed in men (47.0%) than in women (22.7%), consistent with results reported in other published studies (51, 52). Research revealed that women tend to manifest emotions such as anxiety or depression more frequently, while men, under chronic stress conditions, are more likely to exhibit an elevated incidence of alcohol consumption and an increased risk of hypertension and MetS (53–55). The mechanisms underlying the relationship between psychosocial stress and hypertension are diverse and complex (56). Furthermore, many cross-sectional and cohort studies have reported that psychological stress is not involved in the progression of hypertension. Therefore, based on current evidence, we cannot conclude that psychological stress is a risk factor for hypertension in the general population (57, 58).

Regarding the relationship between psychological stress and overweight or obesity, hyperglycemia, and dyslipidemia, no significant association was observed in our cross-sectional and MR results, supporting the findings of previous cross-sectional and cohort studies (59, 60). However, several publications that additionally adjusted for the confounding effects of dietary behavior showed a significant relationship between psychological stress and the aforementioned factors (61, 62). Research related to behavioral psychology has indicated that high-income populations respond to high levels of psychological stress through physical activity, whereas some low-income populations are more likely to cope with it through compensatory eating (63). Due to limitations in data collection for this project, we did not include dietary habits as covariates in the regression analysis. Additionally, it is worth noting that most of the study population consisted of high-income populations, which could be one possible reason for the non-significant results. Furthermore, the range of CPSS scores in this current study cannot reflect the psychological stress of highly stressed individuals, potentially explaining the lack of a significant correlation (64).

### Strengths and limitations

This study had several limitations that should be considered. Firstly, compared to clinical diagnosis, the self-reported questionnaires (i.e., SDS, SAS, and PSQI) used in this cross-sectional study provided limited evidence. Secondly, due to limitations in data collection for this project, we did not include dietary habits as covariates in the regression analysis. Additionally, it is worth noting that most of the study populations consisted of high-income populations, which could be one possible reason for the nonsignificant results. Furthermore, the range of CPSS scores in this current study cannot reflect the psychological stress of highly stressed individuals, potentially explaining the lack of a significant correlation. Moreover, the cross-sectional study design cannot avoid the influence of traditional confounding factors and inverse causal associations. The reason for the lack of detailed demographic information is that we did not perform subgroup analyses in the MR analyses. Finally, owing to data limitations, the current observational study in the Chinese population and the MR study in the European population both constrain the generalizability of our study results. The strengths of this study are as follows: the confounding effects of depression, anxiety, and sleep quality, which have rarely been accounted for in previous epidemiological studies, were adjusted using regression analysis in the current cross-sectional study (9). In MR analysis, we investigated the causal relationship between psychological stress and MetS and its components from a genetic perspective.

## Conclusion

In conclusion, our findings did not indicate a significant association between psychological stress and MetS. However, we observed associations between psychological stress and hypertension, with evidence that individuals with hypertension may be more susceptible to psychological stress. These findings may have implications for targeting factors related to hypertension and psychological stress in interventions aimed at improving mental and metabolic health. The relationship between psychological stress and MetS and its components requires further study and careful interpretation.

## Data availability statement

The raw data supporting the conclusions of this article will be made available by the authors, without undue reservation.

## Ethics statement

The studies involving humans were approved by Medical Ethics Committee of Chinese People’s Liberation Army General Hospital (S2019-131-01). The studies were conducted in accordance with the local legislation and institutional requirements. The participants provided their written informed consent to participate in this study. Written informed consent was obtained from the individual(s) for the publication of any potentially identifiable images or data included in this article.

## Author contributions

YN and WG contributed to the design and supervision of this study. CL and TT participated in the design and planning process. YT, HZ, and HXL collected and compiled the data. CL and HML analyzed the data. CL and TT wrote the first draft of the manuscript. YN, XL, and TT revised the manuscript. All authors contributed to the article and approved the submitted version.
