# Supplementary material for: The association of psychological stress with metabolic syndrome and its components: cross-sectional and bidirectional two-sample Mendelian randomization analyses
Source: Front Endocrinol (Lausanne). 2023 Dec 8;14:1212647. doi: 10.3389/fendo.2023.1212647 (PMC10749192; doi:10.3389/fendo.2023.1212647)
Supplement: Supplementary file 1 [file DataSheet_1.docx]

A list of Supporting Materials

Supplementary Table 1 Details of the data sources used in this bidirectional two-sample MR study……………………………………………….…………3

Supplementary Table 2 Characteristics of selected SNPs for psychological stress (stress-related disorders)……………….…………………………………4
Supplementary Table 3 Forward causal relationships of the psychological stress with MetS and its components using MR………………………………6

Supplementary Table 4 The R^2^ and F-statistics for the genetic instruments and power for MR…………………………………………………………………9

Supplementary Table 5 Characteristics of selected SNPs for MetS according to psychological stress………………………………………………………10
Supplementary Table 6 Characteristics of selected SNPs for hypertension according to psychological stress………………………………………………13
Supplementary Table 7 Characteristics of selected SNPs for overweight according to psychological stress………………………………………………16

Supplementary Table 8 Characteristics of selected SNPs for obesity according to psychological stress……………………………………………………17
Supplementary Table 9 Characteristics of selected SNPs for BMI according to psychological stress…………………………………………………………18

Supplementary Table 10 Characteristics of selected SNPs for hyperlipidemia according to psychological stress………………………………………20
Supplementary Table 11 Characteristics of selected SNPs for HDL-C according to psychological stress……………………………………………………21
Supplementary Table 12 Characteristics of selected SNPs for TG according to psychological stress…………………………………………………………24
Supplementary Table 13 Characteristics of selected SNPs for FBG according to psychological stress………………………………………………………26

Supplementary Table 14 Reverse causal relationships of MetS and its components with psychological stress using MR………………………………30

Supplementary Figure 1 Diagram of the hypothesis of instrumental variables in Mendelian randomization study………………………………………33

Supplementary Figure 2 The forest plots of the association between genetically predicted psychological stress on MetS and its components in MR analysis……………………………………………………………………………………………………………………………………………………………………......…34

Supplementary Figure 3 The funnel plots of the association between genetically predicted psychological stress on MetS and its components in MR analysis...…………………………………………………………………………………………………………………………………………………………...……………35

Supplementary Figure 4 The leave-one-out analysis of the association between genetically predicted psychological stress on MetS and its components in MR analysis…………………………………………………………………………………………………………………………………………………36

Supplementary Figure 5 The scatter plots of the association between genetically predicted psychological stress on MetS and its components in MR analysis……………………………………………………………………………………….………………………………………………………………………………….37

Supplementary Figure 6 The forest plots of the association between genetically predicted MetS and its components on psychological stress in MR analysis………………………………………………………………………………………………………………….…………………………….…………………………38

Supplementary Figure 7 The funnel plots of the association between genetically predicted MetS and its components on psychological stress in MR analysis………………………………………………….………………………………………….……………………………………………………………………………39

Supplementary Figure 8 The leave-one-out analysis of the association between genetically predicted MetS and its components on psychological stress in MR analysis………….…………….……………….………………………………….……………….……………………………………………………………40

Supplementary Figure 9 The scatter plots of the association between genetically predicted MetS and its components on psychological stress in MR analysis…………………………………………………………………….……………….……………………………………………………………………………………41

Supplementary Table 1 Details of the data sources used in this bidirectional two-sample MR study.

| Factors | Exposure | | | | Outcome | | |
| --- | --- | --- | --- | --- | --- | --- | --- |
|  | GWAS ID/PMID | Samples | Consortium or cohorts | No. of SNPs | Consortium or cohorts | Samples | Sources |
| Psychological stress, stress-related disorders^*^ | GWAS ID:  finn-b-F5_NEUROTIC | 218,792 | a GWAS in FinnGen biobank | 40 | FinnGen biobank | 218,792 | https://gwas.mrcieu.ac.uk/ |
| MetS | PMID: 31589552 | 291,107 | a GWAS in UK Biobank | 93 | UK Biobank | 291,107 | https://wwwebi.ac.uk/gwas/downloads/summary-statistics |
| Hypertension | GWAS ID: ukb-b-12493 | 463,010 | a GWAS in MRC-IEU UK Biobank | 71 | MRC-IEU UK Biobank | 463,010 | https://gwas.mrcieu.ac.uk/ |
| Overweight | GWAS ID: ieu-a-93 | 158,855 | a GWAS in GIANT | 14 | GIANT | 158,855 | https://gwas.mrcieu.ac.uk/ |
| Obesity | GWAS ID: ukb-b-15541 | 463,010 | a GWAS in GIANT | 13 | MRC-IEU UK Biobank | 463,010 | https://gwas.mrcieu.ac.uk/ |
| BMI | GWAS ID: ieu-a-974 | 171,977 | a GWAS in GIANT | 37 | GIANT | 171,977 | https://gwas.mrcieu.ac.uk/ |
| Hyperlipidaemia | GWAS ID: ukb-b-17462 | 463,010 | a GWAS in MRC-IEU UK Biobank | 11 | UK Biobank | 463,010 | https://gwas.mrcieu.ac.uk/ |
| HDL-C | PMID: 24097068 | 188,577 | GLGC | 71 | Japan Biobank | 70,657 | https://gwas.mrcieu.ac.uk/ |
| TG | PMID: 24097068 | 188,577 | GLGC | 31 | UK Biobank | 441,016 | https://gwas.mrcieu.ac.uk/ |
| FBG | PMID: 34059833 | 281,416 | the Meta-Analyses of MAGIC | 119 | MAGIC | 281,416 | https://magicinvestigators.org/ |

Note: MR, mendelian randomization; GWAS, genome-wide association studies; SNPs, single nucleotide polymorphisms; MetS, metabolic syndrome; BMI, body mass index; HDL-C, high-density lipoprotein cholesterol; TG, triglycerides; FBG, fasting blood glucose; GIANT, Genetic Investigation of ANthropometric Traits; MAGIC, Glucose and Insulin-related traits Consortium; GLGC, Global Lipids Genetics Consortium. ^*^ Considering that there are currently no published data on GWAS of psychological stress, we employed a bidirectional two-sample MR analysis, which included GWAS results of stress-related disorders, MetS, and its components, to validate our cross-sectional findings.

Supplementary Table 2 Characteristics of selected SNPs for psychological stress (stress-related disorders).

| SNP | Trait ^*^ | Chr | Pos. | Effect allele | Other allele | EAF | Beta | SE | *P* value | R^2^ | *F* statistic |
| --- | --- | --- | --- | --- | --- | --- | --- | --- | --- | --- | --- |
| rs1933530 | Stress-related disorders | 1 | 2.46E+08 | G | T | 0.2547 | -0.0613 | 0.0129 | 2.17E-06 | 0.001427 | 311.6868 |
| rs143927812 | Stress-related disorders | 1 | 1.51E+08 | A | G | 0.006512 | 0.3416 | 0.073 | 2.91E-06 | 0.00151 | 329.8481 |
| rs2224257 | Stress-related disorders | 1 | 70205848 | C | G | 0.1925 | 0.0649 | 0.0143 | 5.61E-06 | 0.001309 | 286.1219 |
| rs116613090 | Stress-related disorders | 1 | 2.08E+08 | T | A | 0.02998 | 0.1613 | 0.0331 | 1.11E-06 | 0.001513 | 330.5832 |
| rs61820769 | Stress-related disorders | 1 | 2.14E+08 | T | C | 0.03743 | -0.1532 | 0.0301 | 3.54E-07 | 0.001691 | 369.3959 |
| rs541301 | Stress-related disorders | 1 | 37250054 | C | T | 0.3246 | 0.0531 | 0.012 | 9.66E-06 | 0.001236 | 270.1586 |
| rs779976 | Stress-related disorders | 2 | 1.25E+08 | T | A | 0.4032 | 0.0511 | 0.0115 | 8.25E-06 | 0.001257 | 274.6012 |
| rs150473809 | Stress-related disorders | 3 | 65137708 | A | C | 0.01077 | 0.2425 | 0.0545 | 8.66E-06 | 0.001253 | 273.8101 |
| rs7681873 | Stress-related disorders | 4 | 93488944 | T | C | 0.001328 | 0.8786 | 0.1969 | 8.12E-06 | 0.002048 | 447.0650 |
| rs4975306 | Stress-related disorders | 4 | 1.43E+08 | G | A | 0.1935 | 0.063 | 0.0142 | 9.64E-06 | 0.001239 | 270.6983 |
| rs11729045 | Stress-related disorders | 4 | 1.12E+08 | A | C | 0.07413 | -0.118 | 0.0218 | 6.04E-08 | 0.001911 | 417.3829 |
| rs13190528 | Stress-related disorders | 5 | 60906140 | G | A | 0.06249 | 0.1189 | 0.0234 | 3.83E-07 | 0.001656 | 361.8159 |
| rs72996580 | Stress-related disorders | 6 | 1.04E+08 | A | G | 0.1128 | 0.0849 | 0.0178 | 1.91E-06 | 0.001443 | 315.1929 |
| rs118078990 | Stress-related disorders | 6 | 1.71E+08 | T | C | 0.05041 | -0.1187 | 0.026 | 4.99E-06 | 0.001349 | 294.7308 |
| rs11965537 | Stress-related disorders | 6 | 94138233 | C | G | 0.02245 | 0.1764 | 0.0381 | 3.59E-06 | 0.001366 | 298.4121 |
| rs13191326 | Stress-related disorders | 6 | 27031029 | G | A | 0.07441 | -0.105 | 0.0222 | 2.17E-06 | 0.001519 | 331.7615 |
| rs12208240 | Stress-related disorders | 6 | 45501937 | A | G | 0.05091 | -0.1287 | 0.0263 | 9.72E-07 | 0.001601 | 349.6466 |
| rs78840759 | Stress-related disorders | 7 | 82762801 | C | T | 0.02109 | -0.1902 | 0.04 | 2.01E-06 | 0.001494 | 326.3237 |
| rs60738304 | Stress-related disorders | 7 | 1E+08 | C | A | 0.7113 | -0.0649 | 0.0124 | 1.79E-07 | 0.00173 | 377.8283 |
| rs533968 | Stress-related disorders | 8 | 1.03E+08 | G | A | 0.06352 | 0.1033 | 0.0231 | 7.72E-06 | 0.00127 | 277.4057 |
| rs116905709 | Stress-related disorders | 9 | 1.17E+08 | A | G | 0.02179 | 0.1855 | 0.0389 | 1.92E-06 | 0.001467 | 320.4772 |
| rs145070376 | Stress-related disorders | 9 | 1.4E+08 | C | G | 0.006683 | 0.3538 | 0.0735 | 1.47E-06 | 0.001662 | 363.0029 |
| rs77506328 | Stress-related disorders | 11 | 45282796 | A | G | 0.00368 | 0.4233 | 0.0947 | 7.83E-06 | 0.001314 | 287.0977 |
| rs113164726 | Stress-related disorders | 11 | 89657005 | C | T | 0.04926 | 0.1181 | 0.026 | 5.46E-06 | 0.001306 | 285.4603 |
| rs11245950 | Stress-related disorders | 11 | 1094629 | C | T | 0.4516 | -0.0507 | 0.0113 | 7.73E-06 | 0.001273 | 278.2092 |
| rs2193743 | Stress-related disorders | 12 | 1.09E+08 | G | A | 0.2629 | 0.0639 | 0.0129 | 6.59E-07 | 0.001583 | 345.6912 |
| rs10492174 | Stress-related disorders | 12 | 97489165 | A | G | 0.09227 | -0.089 | 0.0195 | 5.17E-06 | 0.001327 | 289.9199 |
| rs1357251 | Stress-related disorders | 12 | 24375601 | C | T | 0.0345 | 0.1443 | 0.0312 | 3.85E-06 | 0.001387 | 303.0809 |
| rs58328352 | Stress-related disorders | 13 | 57086974 | T | A | 0.1729 | -0.0663 | 0.0149 | 9.03E-06 | 0.001257 | 274.7206 |
| rs12886630 | Stress-related disorders | 14 | 21468955 | C | T | 0.405 | 0.0512 | 0.0115 | 8.61E-06 | 0.001263 | 276.0707 |
| rs67774492 | Stress-related disorders | 15 | 70131310 | G | A | 0.1423 | -0.0737 | 0.0163 | 6.12E-06 | 0.001326 | 289.7054 |
| rs144051380 | Stress-related disorders | 16 | 23593287 | A | G | 0.04991 | 0.1345 | 0.0262 | 2.75E-07 | 0.001716 | 374.7215 |
| rs17138385 | Stress-related disorders | 16 | 5736726 | T | C | 0.1507 | 0.0717 | 0.0159 | 6.27E-06 | 0.001316 | 287.5400 |
| rs55776706 | Stress-related disorders | 16 | 73705972 | C | T | 0.2615 | 0.0602 | 0.0128 | 2.50E-06 | 0.0014 | 305.8189 |
| rs78531070 | Stress-related disorders | 17 | 5566962 | A | G | 0.06101 | 0.1081 | 0.0239 | 6.30E-06 | 0.001339 | 292.5425 |
| rs7237747 | Stress-related disorders | 18 | 52476108 | G | C | 0.4507 | 0.0558 | 0.0113 | 8.34E-07 | 0.001542 | 336.7852 |
| rs62205564 | Stress-related disorders | 20 | 59101886 | T | C | 0.02872 | -0.155 | 0.034 | 5.33E-06 | 0.00134 | 292.8648 |
| rs73913948 | Stress-related disorders | 20 | 54330860 | A | G | 0.1105 | -0.0888 | 0.0183 | 1.22E-06 | 0.00155 | 338.6241 |
| rs71317018 | Stress-related disorders | 22 | 37355886 | G | A | 0.01387 | 0.2339 | 0.0504 | 3.43E-06 | 0.001497 | 326.9476 |
| rs2913 | Stress-related disorders | 22 | 18982097 | A | G | 0.6527 | 0.0524 | 0.0118 | 9.86E-06 | 0.001245 | 272.0179 |

Note, Chr, chromosome; EAF, Effect allele frequency; Pos, position; SE, standard error; SNP, single-nucleotide polymorphism. The threshold was set at *P* < 1×10^-5^. ^*^Considering that there are currently no published data on Genome-Wide Association Studies (GWAS) of psychological stress, we utilized the GWAS results of stress-related disorders for validation.

Supplementary Table 3 Forward causal relationships of psychological stress with MetS and its components performed using MR

| Exposure | Outcome | nSNPs | Method | OR (95%CI) | *P* | Q pval | Intercept pval | Global *P* |
| --- | --- | --- | --- | --- | --- | --- | --- | --- |
| Psychological stress | MetS | 36 | IVW | 0.989 (0.853, 1.146) | 0.226 | 0.023 |  |  |
|  |  | 36 | MR Egger | 0.963 (0.625, 1.483) | 0.356 |  | 0.689 |  |
|  |  | 36 | MR-PRESSO | 0.992 (0.973, 1.011) | 0.412 |  |  | 0.151 |
|  |  | 36 | Weighted median | 0.959 (0.835, 1.101) | 0.065 |  |  |  |
|  |  | 36 | Simple mode | 0.912 (0.735, 1.132) | 0.144 |  |  |  |
|  |  | 36 | Weighted mode | 0.913 (0.748, 1.113) | 0.085 |  |  |  |
| Psychological stress | Hypertension | 36 | IVW | 1.001 (0.996, 1.004) | 0.929 | 0.949 |  |  |
|  |  | 36 | MR Egger | 0.999 (0.992, 1.007) | 0.943 |  | 0.894 |  |
|  |  | 36 | MR-PRESSO | 2.389 (1.241, 4.600) | 0.011 |  |  | 0.945 |
|  |  | 36 | Weighted median | 0.999 (0.993, 1.004) | 0.661 |  |  |  |
|  |  | 36 | Simple mode | 0.997 (0.986, 1.008) | 0.634 |  |  |  |
|  |  | 36 | Weighted mode | 0.997 (0.988, 1.006) | 0.504 |  |  |  |
| Psychological stress | Overweight | 17 | IVW | 0.951 (0.879, 1.030) | 0.217 | 0.918 |  |  |
|  |  | 17 | MR Egger | 0.948 (0.789, 1.140) | 0.581 |  | 0.971 |  |
|  |  | 17 | MR-PRESSO | 0.992 (0.975, 1.009) | 0.355 |  |  | 0.798 |
|  |  | 17 | Weighted median | 0.947 (0.851, 1.055) | 0.323 |  |  |  |
|  |  | 17 | Simple mode | 1.056 (0.872, 1.278) | 0.586 |  |  |  |
|  |  | 17 | Weighted mode | 1.037 (0.880, 1.222) | 0.669 |  |  |  |
| Psychological stress | Obesity | 21 | IVW | 0.999 (0.998, 1.002) | 0.957 | 0.110 |  |  |
|  |  | 21 | MR Egger | 0.997 (0.990, 1.004) | 0.412 |  | 0.401 |  |
|  |  | 21 | MR-PRESSO | 0.999 (0.998, 1.001) | 0.165 |  |  | 0.368 |
|  |  | 21 | Weighted median | 1.001 (0.998, 1.003) | 0.924 |  |  |  |
|  |  | 21 | Simple mode | 1.001 (0.996, 1.007) | 0.616 |  |  |  |
|  |  | 21 | Weighted mode | 1.002 (0.996, 1.008) | 0.569 |  |  |  |
| Psychological stress | BMI | 18 | IVW | 0.982 (0.945, 1.021) | 0.358 | 0.800 |  |  |
|  |  | 18 | MR Egger | 1.031 (0.948, 1.122) | 0.484 |  | 0.226 |  |
|  |  | 18 | MR-PRESSO | 0.994 (0.985, 1.004) | 0.241 |  |  | 0.878 |
|  |  | 18 | Weighted median | 1.009 (0.958, 1.062) | 0.746 |  |  |  |
|  |  | 18 | Simple mode | 1.009 (0.920, 1.106) | 0.848 |  |  |  |
|  |  | 18 | Weighted mode | 1.017 (0.954, 1.084) | 0.614 |  |  |  |
| Psychological stress | Hyperlipidaemia | 15 | IVW | 0.999 (0.998, 1.002) | 0.943 | 0.320 |  |  |
|  |  | 15 | MR Egger | 0.994 (0.985, 1.003) | 0.231 |  | 0.228 |  |
|  |  | 15 | MR-PRESSO | 0.999 (0.998, 1.001) | 0.250 |  |  | 0.645 |
|  |  | 15 | Weighted median | 0.999 (0.997, 1.002) | 0.761 |  |  |  |
|  |  | 15 | Simple mode | 0.999 (0.995, 1.004) | 0.808 |  |  |  |
|  |  | 15 | Weighted mode | 0.999 (0.996, 1.003) | 0.707 |  |  |  |
| Psychological stress | HDL-C | 15 | IVW | 1.036 (0.981, 1.094) | 0.208 | 0.269 |  |  |
|  |  | 15 | MR Egger | 0.764 (0.590, 0.990) | 0.063 |  | 0.035 |  |
|  |  | 15 | MR-PRESSO | 1.008 (0.993, 1.022) | 0.308 |  |  | 0.511 |
|  |  | 15 | Weighted median | 0.975 (0.908, 1.048) | 0.497 |  |  |  |
|  |  | 15 | Simple mode | 0.967 (0.858, 1.090) | 0.586 |  |  |  |
|  |  | 15 | Weighted mode | 0.972 (0.876, 1.079) | 0.617 |  |  |  |
| Psychological stress | TG | 38 | IVW | 0.999 (0.982, 1.016) | 0.902 | 1.361e-06 |  |  |
|  |  | 38 | MR Egger | 1.003 (0.969, 1.038) | 0.876 |  | 0.801 |  |
|  |  | 38 | MR-PRESSO | 1.001 (0.994, 1.008) | 0.790 |  |  | 0.007 |
|  |  | 38 | Weighted median | 0.991 (0.975, 1.008) | 0.293 |  |  |  |
|  |  | 38 | Simple mode | 0.989 (0.957, 1.021) | 0.485 |  |  |  |
|  |  | 38 | Weighted mode | 0.988 (0.962, 1.014) | 0.350 |  |  |  |
| Psychological stress | FBG | 38 | IVW | 1.002 (0.991, 1.013) | 0.739 | 0.721 |  |  |
|  |  | 38 | MR Egger | 1.003 (0.980, 1.027) | 0.778 |  | 0.892 |  |
|  |  | 38 | MR-PRESSO | 1.006 (1.002, 1.010) | 0.007 |  |  | 0.819 |
|  |  | 38 | Weighted median | 1.008 (0.991, 1.025) | 0.370 |  |  |  |
|  |  | 38 | Simple mode | 1.020 (0.981, 1.060) | 0.325 |  |  |  |
|  |  | 38 | Weighted mode | 1.012 (0.983, 1.043) | 0.427 |  |  |  |

Note: MetS, metabolic syndrome; MR: Mendelian randomization; nSNPs: number of single-nucleotide polymorphisms; OR: odds ratio; CI: confidence interval; Q pval: P value of the Cochran Q statistic; IVW: inverse-variance weighted; MR-PRESSO: MR pleiotropy residual sum and outlier; BMI: body mass index; FBG: fasting blood-glucose; HDL-C: high-density lipoprotein cholesterol; TG, triglycerides.

Supplementary Table 4 R^2^ and *F*-statistics for the genetic instruments and power for MR

| Exposure | | Outcome | | nSNPs | R^2^ | *F*-statistics | Power |
| --- | --- | --- | --- | --- | --- | --- | --- |
| Trait ^*^ | Sample size | Trait ^*^ | Sample size |  |  |  |  |
| The forward MR analysis |  |  |  |  |  |  |  |
| Stress-related disorders | 218,792 | MetS | 291,107 | 36 | 0.0510 | 11171.549 | 99.0% |
| Stress-related disorders | 218,792 | Hypertension | 463,010 | 36 | 0.0514 | 11261.264 | 100.0% |
| Stress-related disorders | 218,792 | Overweight | 158,855 | 17 | 0.0274 | 6003.875 | 100.0% |
| Stress-related disorders | 218,792 | Obesity | 463,010 | 21 | 0.0296 | 6474.438 | 100.0% |
| Stress-related disorders | 218,792 | BMI | 171,977 | 18 | 0.0250 | 5478.292 | 78.0% |
| Stress-related disorders | 218,792 | Hyperlipidaemia | 463,010 | 15 | 0.0204 | 4464.888 | 100.0% |
| Stress-related disorders | 218,792 | HDL-C | 441,016 | 15 | 0.0200 | 4451.231 | 100.0% |
| Stress-related disorders | 218,792 | TG | 441,016 | 38 | 0.0550 | 12080.810 | 93.0% |
| Stress-related disorders | 218,792 | FBG | 281,416 | 38 | 0.0540 | 11906.41 | 75.0% |
| The reverse MR analysis |  |  |  |  |  |  |  |
| MetS | 291,107 | Stress-related disorders | 218,792 | 68 | 0.1334 | 39099.960 | 97.0% |
| Hypertension | 463,010 | Stress-related disorders | 218,792 | 66 | 0.0010 | 335.550 | 100.0% |
| Overweight | 158,855 | Stress-related disorders | 218,792 | 14 | 0.0343 | 5479.997 | 100.0% |
| Obesity | 463,010 | Stress-related disorders | 218,792 | 13 | 8.13E-06 | 3.766 | 65.0% |
| BMI | 171,977 | Stress-related disorders | 218,792 | 37 | 0.0195 | 3353.618 | 65.0% |
| Hyperlipidaemia | 463,010 | Stress-related disorders | 218,792 | 11 | 4.68E-06 | 2.165 | 65.0% |
| HDL-C | 188,577 | Stress-related disorders | 218,792 | 69 | 0.0730 | 13904.160 | 96.0% |
| TG | 188,577 | Stress-related disorders | 218,792 | 37 | 0.1668 | 34603.830 | 93.0% |
| FBG | 281,416 | Stress-related disorders | 218,792 | 94 | 0.0204 | 5731.313 | 98.0% |

Note: MR, Mendelian randomization; nSNPs, number of single-nucleotide polymorphisms; MetS, metabolic syndrome; BMI, body mass index; FBG, fasting blood-glucose; HDL-C, high-density lipoprotein cholesterol; TG, triglycerides. ^*^ Considering that there are currently no published data on GWAS of psychological stress, we employed a bidirectional two-sample MR analysis, which included GWAS results of stress-related disorders, MetS, and its components, to validate our cross-sectional findings.

Supplementary Table 5 Characteristics of selected SNPs for MetS according to psychological stress.

| SNP | Trait | Chr | Pos. | Effect allele | Other allele | EAF | Beta | SE | *P* value | R^2^ | *F* statistic |
| --- | --- | --- | --- | --- | --- | --- | --- | --- | --- | --- | --- |
| rs10913469 | MetS | 1 | 1.78E+08 | C | T | 0.207314 | 0.048264 | 0.007908 | 2E-09 | 0.000766 | 223.044 |
| rs11206374 | MetS | 1 | 39582337 | A | G | 0.225477 | 0.065077 | 0.007653 | 4E-17 | 0.001479 | 431.2346 |
| rs12752223 | MetS | 1 | 93371576 | T | C | 0.396475 | 0.037483 | 0.006888 | 2E-08 | 0.000672 | 195.8637 |
| rs638714 | MetS | 1 | 62440818 | T | G | 0.347785 | 0.051495 | 0.006888 | 1E-13 | 0.001203 | 350.6234 |
| rs1009360 | MetS | 2 | 65048915 | C | T | 0.415241 | 0.043589 | 0.006378 | 5E-11 | 0.000923 | 268.8472 |
| rs10187501 | MetS | 2 | 1.65E+08 | G | A | 0.346097 | 0.046559 | 0.006888 | 2E-11 | 0.000981 | 285.908 |
| rs12472667 | MetS | 2 | 1.71E+08 | G | C | 0.371305 | 0.03749 | 0.006888 | 3E-08 | 0.000656 | 191.1414 |
| rs1260326 | MetS | 2 | 27508073 | T | C | 0.392938 | 0.053697 | 0.006633 | 7E-16 | 0.001376 | 400.9925 |
| rs2138161 | MetS | 2 | 2.26E+08 | T | C | 0.351852 | 0.075241 | 0.006888 | 8E-28 | 0.002582 | 753.6146 |
| rs62107261 | MetS | 2 | 422144 | C | T | 0.048257 | 0.089776 | 0.015561 | 5E-09 | 0.00074 | 215.6747 |
| rs673548 | MetS | 2 | 21014672 | T | C | 0.204049 | 0.09779 | 0.008163 | 2E-32 | 0.003106 | 907.0675 |
| rs7563362 | MetS | 2 | 620297 | A | G | 0.142333 | 0.061357 | 0.009439 | 1E-10 | 0.000919 | 267.8094 |
| rs10049088 | MetS | 3 | 1.57E+08 | T | C | 0.386891 | 0.044322 | 0.006888 | 5E-11 | 0.000932 | 271.5462 |
| rs61789601 | MetS | 3 | 1.36E+08 | T | C | 0.202909 | 0.068372 | 0.008163 | 8E-17 | 0.001512 | 440.8582 |
| rs13107325 | MetS | 4 | 1.02E+08 | T | C | 0.074856 | 0.07609 | 0.012245 | 4E-10 | 0.000802 | 233.6262 |
| rs73123462 | MetS | 4 | 36075982 | T | C | 0.015597 | 0.149549 | 0.02551 | 5E-09 | 0.000687 | 200.0557 |
| rs2307111 | MetS | 5 | 75707853 | C | T | 0.393101 | 0.036672 | 0.006633 | 5E-08 | 0.000642 | 186.9116 |
| rs10945840 | MetS | 6 | 1.63E+08 | C | G | 0.332614 | 0.038293 | 0.006888 | 4E-08 | 0.000651 | 189.6362 |
| rs11751347 | MetS | 6 | 1.61E+08 | T | C | 0.102714 | 0.062488 | 0.010459 | 5E-09 | 0.00072 | 209.6714 |
| rs11754773 | MetS | 6 | 34609480 | G | A | 0.094228 | 0.089294 | 0.010969 | 5E-16 | 0.001361 | 396.7475 |
| rs5021727 | MetS | 6 | 32610856 | G | A | 0.461566 | 0.037134 | 0.006633 | 2E-08 | 0.000685 | 199.6539 |
| rs577721086 | MetS | 6 | 1.27E+08 | C | T | 0.05096 | 0.090094 | 0.014796 | 9E-10 | 0.000785 | 228.7298 |
| rs632057 | MetS | 6 | 1.4E+08 | T | G | 0.372799 | 0.048679 | 0.006888 | 5E-13 | 0.001108 | 322.9444 |
| rs76376137 | MetS | 6 | 34205553 | G | T | 0.050549 | 0.082033 | 0.014796 | 1E-09 | 0.000646 | 188.1575 |
| rs9378248 | MetS | 6 | 31358512 | A | G | 0.339668 | 0.044068 | 0.006888 | 2E-10 | 0.000871 | 253.8223 |
| rs998584 | MetS | 6 | 43790159 | A | C | 0.481524 | 0.070685 | 0.006633 | 4E-27 | 0.002495 | 728.0615 |
| rs10260148 | MetS | 7 | 1.31E+08 | T | C | 0.27901 | 0.052129 | 0.007143 | 7E-13 | 0.001093 | 318.6086 |
| rs12056034 | MetS | 7 | 73464315 | G | A | 0.124753 | 0.096837 | 0.010204 | 8E-22 | 0.002048 | 597.3512 |
| rs1534696 | MetS | 7 | 26357619 | C | A | 0.460436 | 0.042436 | 0.006378 | 9E-11 | 0.000895 | 260.7076 |
| rs56282717 | MetS | 7 | 1.51E+08 | A | G | 0.24425 | 0.046096 | 0.007653 | 2E-09 | 0.000784 | 228.5426 |
| rs10954772 | MetS | 8 | 31006422 | T | C | 0.313991 | 0.039198 | 0.007143 | 3E-08 | 0.000662 | 192.8195 |
| rs28597716 | MetS | 8 | 20079176 | G | A | 0.189687 | 0.152184 | 0.007653 | 1E-24 | 0.00712 | 2087.426 |
| rs2980888 | MetS | 8 | 1.25E+08 | T | C | 0.299847 | 0.095952 | 0.007143 | 4E-42 | 0.003866 | 1129.703 |
| rs3808439 | MetS | 8 | 1.16E+08 | A | G | 0.447923 | 0.038346 | 0.006633 | 5E-09 | 0.000727 | 211.8517 |
| rs3844510 | MetS | 8 | 20003850 | C | A | 0.26399 | 0.191134 | 0.007653 | 1E-138 | 0.014196 | 4192.145 |
| rs4921913 | MetS | 8 | 18414867 | C | T | 0.221605 | 0.042644 | 0.007908 | 5E-08 | 0.000627 | 182.7468 |
| rs9987289 | MetS | 8 | 9325848 | A | G | 0.091975 | 0.087388 | 0.010969 | 3E-15 | 0.001276 | 371.7982 |
| rs11789603 | MetS | 9 | 1.05E+08 | T | C | 0.107873 | 0.058891 | 0.010714 | 4E-08 | 0.000668 | 194.4496 |
| rs10822155 | MetS | 10 | 63311455 | A | C | 0.41581 | 0.051119 | 0.006633 | 2E-14 | 0.00127 | 370.0297 |
| rs563296 | MetS | 10 | 98012647 | G | A | 0.44029 | 0.04194 | 0.006633 | 2E-10 | 0.000867 | 252.5882 |
| rs1535 | MetS | 11 | 61830500 | G | A | 0.347774 | 0.079213 | 0.006888 | 3E-31 | 0.002847 | 830.9986 |
| rs2306363 | MetS | 11 | 65638129 | T | G | 0.206589 | 0.048276 | 0.008163 | 3E-09 | 0.000764 | 222.5776 |
| rs35661464 | MetS | 11 | 65061370 | T | C | 0.252033 | 0.04638 | 0.007398 | 5E-10 | 0.000811 | 236.2825 |
| rs56133711 | MetS | 11 | 27701787 | A | G | 0.262839 | 0.04433 | 0.007398 | 2E-09 | 0.000762 | 221.8513 |
| rs7124681 | MetS | 11 | 47508395 | A | C | 0.408583 | 0.055786 | 0.006633 | 3E-17 | 0.001504 | 438.4882 |
| rs9332817 | MetS | 11 | 1.18E+08 | C | G | 0.026501 | 0.115795 | 0.020918 | 4E-08 | 0.000692 | 201.5353 |
| rs964184 | MetS | 11 | 1.17E+08 | G | C | 0.132408 | 0.252628 | 0.010204 | 7E-167 | 0.014663 | 4331.992 |
| rs56959712 | MetS | 12 | 1.23E+08 | T | G | 0.210819 | 0.047049 | 0.008163 | 6E-09 | 0.000737 | 214.5761 |
| rs1023193 | MetS | 15 | 41563538 | T | G | 0.305574 | 0.045397 | 0.007143 | 2E-10 | 0.000875 | 254.8334 |
| rs139974673 | MetS | 15 | 43735687 | C | T | 0.025125 | 0.160596 | 0.020408 | 1E-15 | 0.001263 | 368.2631 |
| rs261290 | MetS | 15 | 58386521 | T | C | 0.347343 | 0.077956 | 0.007143 | 2E-29 | 0.002755 | 804.3109 |
| rs11075253 | MetS | 16 | 15054789 | A | C | 0.297121 | 0.046879 | 0.007143 | 7E-11 | 0.000918 | 267.4517 |
| rs247617 | MetS | 16 | 56956804 | A | C | 0.324218 | 0.196432 | 0.007653 | 2E-166 | 0.016908 | 5006.726 |
| rs3814883 | MetS | 16 | 29983601 | T | C | 0.48348 | 0.039392 | 0.006378 | 2E-09 | 0.000775 | 225.7843 |
| rs56094641 | MetS | 16 | 53772541 | G | A | 0.402905 | 0.073677 | 0.006633 | 1E-28 | 0.002612 | 762.2887 |
| rs7188873 | MetS | 16 | 24715743 | A | G | 0.376011 | 0.03863 | 0.006888 | 1E-08 | 0.0007 | 203.9909 |
| rs1143015 | MetS | 17 | 7581888 | A | G | 0.157971 | 0.04914 | 0.008929 | 3E-08 | 0.000642 | 187.1265 |
| rs11655056 | MetS | 17 | 49286745 | C | T | 0.46087 | 0.035993 | 0.006633 | 4E-08 | 0.000644 | 187.5275 |
| rs11871285 | MetS | 17 | 67844693 | T | G | 0.193205 | 0.047341 | 0.008163 | 8E-09 | 0.000699 | 203.5344 |
| rs12945575 | MetS | 17 | 42561053 | T | C | 0.25004 | 0.044779 | 0.007653 | 3E-09 | 0.000752 | 219.08 |
| rs72836561 | MetS | 17 | 43848758 | T | C | 0.032265 | 0.23953 | 0.015306 | 5E-43 | 0.003583 | 1046.752 |
| rs1105654 | MetS | 18 | 49621376 | G | A | 0.345091 | 0.038799 | 0.006888 | 2E-08 | 0.00068 | 198.2124 |
| rs66922415 | MetS | 18 | 60181418 | G | A | 0.234519 | 0.067846 | 0.007653 | 6E-19 | 0.001653 | 481.9037 |
| rs7239575 | MetS | 18 | 23540071 | C | T | 0.49327 | 0.043234 | 0.006633 | 4E-11 | 0.000934 | 272.2688 |
| rs116843064 | MetS | 19 | 8364439 | A | G | 0.019184 | 0.337476 | 0.02551 | 1E-37 | 0.004286 | 1252.999 |
| rs1532127 | MetS | 19 | 47068681 | G | A | 0.314222 | 0.04721 | 0.007143 | 2E-11 | 0.000961 | 279.885 |
| rs483082 | MetS | 19 | 44912921 | T | G | 0.238624 | 0.08993 | 0.007653 | 2E-32 | 0.002939 | 857.9842 |
| rs8121509 | MetS | 20 | 64080700 | C | T | 0.451827 | 0.036069 | 0.006633 | 4E-08 | 0.000644 | 187.7241 |

Note, MetS, metabolic syndrome; Chr, chromosome; EAF, Effect allele frequency; Pos, position; SE, standard error; SNP, single-nucleotide polymorphism. The threshold was set at *P* < 5×10^-8^

Supplementary Table 6 Characteristics of selected SNPs for hypertension according to psychological stress.

| SNP | Trait | Chr | Pos. | Effect allele | Other allele | EAF | Beta | SE | *P* value | R^2^ | *F* statistic |
| --- | --- | --- | --- | --- | --- | --- | --- | --- | --- | --- | --- |
| rs3790604 | hypertension | 1 | 113046879 | A | C | 0.073159 | 0.009082 | 0.001282 | 1.4E-12 | 1.12E-05 | 5.179422 |
| rs17558745 | hypertension | 1 | 218548521 | T | C | 0.311877 | 0.003993 | 0.000722 | 3.2E-08 | 6.85E-06 | 3.169377 |
| rs11801879 | hypertension | 1 | 11928819 | C | T | 0.088418 | -0.00761 | 0.001178 | 1.1E-10 | 9.32E-06 | 4.316959 |
| rs17035646 | hypertension | 1 | 10796547 | A | G | 0.337161 | 0.00607 | 0.000708 | 1E-17 | 1.65E-05 | 7.625427 |
| rs1275985 | hypertension | 2 | 26911745 | T | C | 0.617253 | -0.00622 | 0.000686 | 1.2E-19 | 1.83E-05 | 8.46127 |
| rs1918898 | hypertension | 2 | 188100639 | T | C | 0.356125 | -0.00397 | 0.000697 | 1.3E-08 | 7.22E-06 | 3.340943 |
| rs10804330 | hypertension | 2 | 227185749 | C | T | 0.431675 | -0.00405 | 0.000678 | 2.3E-09 | 8.05E-06 | 3.727417 |
| rs346078 | hypertension | 3 | 11327840 | C | G | 0.378129 | 0.003872 | 0.000688 | 1.8E-08 | 7.05E-06 | 3.265108 |
| rs3821843 | hypertension | 3 | 53558012 | A | G | 0.678977 | 0.004645 | 0.000725 | 1.5E-10 | 9.4E-06 | 4.354596 |
| rs6766859 | hypertension | 3 | 138055136 | T | C | 0.626849 | -0.00404 | 0.000692 | 5.2E-09 | 7.64E-06 | 3.537395 |
| rs2643826 | hypertension | 3 | 27562988 | T | C | 0.45231 | 0.004939 | 0.000671 | 1.8E-13 | 1.21E-05 | 5.595914 |
| rs7685862 | hypertension | 4 | 111389101 | A | C | 0.795291 | -0.00477 | 0.000826 | 7.8E-09 | 7.4E-06 | 3.424178 |
| rs6822044 | hypertension | 4 | 26787745 | G | C | 0.347199 | -0.00421 | 0.000701 | 1.9E-09 | 8.04E-06 | 3.720605 |
| rs13125101 | hypertension | 4 | 81174592 | A | G | 0.29184 | 0.009552 | 0.000734 | 9.7E-39 | 3.77E-05 | 17.46208 |
| rs3796581 | hypertension | 4 | 156642884 | G | A | 0.183958 | -0.00575 | 0.00086 | 2.3E-11 | 9.92E-06 | 4.591292 |
| rs12656497 | hypertension | 5 | 32831939 | C | T | 0.596328 | 0.005379 | 0.000679 | 2.3E-15 | 1.39E-05 | 6.44877 |
| rs6866614 | hypertension | 5 | 131787137 | G | A | 0.576646 | 0.003921 | 0.000679 | 7.8E-09 | 7.51E-06 | 3.475819 |
| rs56273825 | hypertension | 5 | 110840889 | C | T | 0.021882 | -0.01348 | 0.002404 | 2.1E-08 | 7.78E-06 | 3.60099 |
| rs7700842 | hypertension | 5 | 157824183 | C | T | 0.371105 | -0.00692 | 0.000689 | 8.8E-24 | 2.24E-05 | 10.36125 |
| rs4412193 | hypertension | 6 | 26338056 | G | A | 0.366882 | -0.00472 | 0.000692 | 9.3E-12 | 1.03E-05 | 4.792014 |
| rs7763350 | hypertension | 6 | 43349308 | C | A | 0.322191 | 0.004488 | 0.000712 | 2.9E-10 | 8.8E-06 | 4.073268 |
| rs57139556 | hypertension | 6 | 150998511 | G | A | 0.071572 | -0.00773 | 0.001291 | 2.1E-09 | 7.94E-06 | 3.675098 |
| rs1077394 | hypertension | 6 | 31610384 | T | C | 0.672304 | 0.004163 | 0.000709 | 4.4E-09 | 7.64E-06 | 3.535498 |
| rs9375459 | hypertension | 6 | 127147704 | T | C | 0.437127 | 0.006208 | 0.00067 | 2E-20 | 0.000019 | 8.781453 |
| rs55730499 | hypertension | 6 | 161005610 | T | C | 0.079714 | 0.007794 | 0.00123 | 2.4E-10 | 8.91E-06 | 4.12636 |
| rs6961048 | hypertension | 7 | 27328187 | G | C | 0.101304 | 0.006425 | 0.001104 | 5.8E-09 | 7.52E-06 | 3.480182 |
| rs10245376 | hypertension | 7 | 7272368 | T | G | 0.155336 | 0.005806 | 0.00092 | 2.7E-10 | 8.85E-06 | 4.095642 |
| rs3735533 | hypertension | 7 | 27245893 | C | T | 0.926702 | 0.00878 | 0.001276 | 6E-12 | 1.05E-05 | 4.848901 |
| rs3918226 | hypertension | 7 | 150690176 | T | C | 0.081012 | 0.010156 | 0.001238 | 2.4E-16 | 1.54E-05 | 7.110284 |
| rs6991641 | hypertension | 8 | 10586860 | C | G | 0.598329 | -0.00468 | 0.000686 | 9.2E-12 | 1.05E-05 | 4.875863 |
| rs76452347 | hypertension | 9 | 35906471 | T | C | 0.204442 | -0.00514 | 0.000857 | 2.1E-09 | 8.58E-06 | 3.973424 |
| rs35587371 | hypertension | 10 | 21878144 | A | T | 0.303398 | 0.00481 | 0.000725 | 3.2E-11 | 9.78E-06 | 4.528041 |
| rs72831345 | hypertension | 10 | 63518748 | A | G | 0.145089 | -0.00958 | 0.000944 | 3.5E-24 | 2.28E-05 | 10.54358 |
| rs11191559 | hypertension | 10 | 104867686 | T | C | 0.07762 | -0.00772 | 0.001242 | 5.2E-10 | 8.53E-06 | 3.950484 |
| rs12263737 | hypertension | 10 | 96044913 | A | G | 0.271095 | -0.00441 | 0.000749 | 3.8E-09 | 7.68E-06 | 3.558071 |
| rs10749409 | hypertension | 10 | 122976566 | G | C | 0.684325 | -0.00473 | 0.000717 | 4.2E-11 | 9.67E-06 | 4.477436 |
| rs12762222 | hypertension | 10 | 107289232 | C | T | 0.01942 | 0.013488 | 0.002442 | 3.3E-08 | 6.93E-06 | 3.207964 |
| rs740746 | hypertension | 10 | 115792787 | A | G | 0.73319 | 0.00558 | 0.000755 | 1.4E-13 | 1.22E-05 | 5.641342 |
| rs12258967 | hypertension | 10 | 18727959 | G | C | 0.299316 | -0.00504 | 0.000728 | 4.4E-12 | 1.06E-05 | 4.9308 |
| rs55670730 | hypertension | 11 | 43620008 | T | A | 0.110744 | 0.006003 | 0.001072 | 2.1E-08 | 7.1E-06 | 3.285957 |
| rs568546 | hypertension | 11 | 107321156 | T | C | 0.521082 | -0.00523 | 0.000669 | 5.6E-15 | 1.36E-05 | 6.310599 |
| rs11604462 | hypertension | 11 | 65551648 | A | G | 0.343347 | 0.004418 | 0.0007 | 2.8E-10 | 8.8E-06 | 4.075771 |
| rs12360772 | hypertension | 11 | 1899962 | A | G | 0.186914 | 0.0058 | 0.000859 | 1.5E-11 | 1.02E-05 | 4.735051 |
| rs633185 | hypertension | 11 | 100593538 | C | G | 0.715077 | 0.006529 | 0.000741 | 1.3E-18 | 1.74E-05 | 8.043276 |
| rs3184504 | hypertension | 12 | 111884608 | C | T | 0.517267 | -0.00609 | 0.000665 | 5.3E-20 | 1.85E-05 | 8.581878 |
| rs35443 | hypertension | 12 | 115552878 | C | G | 0.381805 | -0.00495 | 0.000685 | 4.6E-13 | 1.16E-05 | 5.361551 |
| rs7297416 | hypertension | 12 | 54443090 | C | A | 0.297749 | -0.00401 | 0.000728 | 3.6E-08 | 6.73E-06 | 3.117241 |
| rs2728624 | hypertension | 12 | 20155052 | A | G | 0.226934 | -0.00459 | 0.000797 | 8.5E-09 | 7.38E-06 | 3.418198 |
| rs8042127 | hypertension | 15 | 41251512 | T | C | 0.479558 | 0.003919 | 0.00067 | 4.8E-09 | 7.67E-06 | 3.549181 |
| rs7497304 | hypertension | 15 | 91429176 | T | G | 0.325747 | 0.005862 | 0.00071 | 1.5E-16 | 1.51E-05 | 6.988579 |
| rs2759315 | hypertension | 15 | 81009646 | A | C | 0.44312 | 0.004714 | 0.000671 | 2.1E-12 | 0.000011 | 5.077764 |
| rs12932686 | hypertension | 16 | 51759252 | C | T | 0.413548 | 0.003897 | 0.000677 | 8.6E-09 | 7.36E-06 | 3.409962 |
| rs77924615 | hypertension | 16 | 20392332 | A | G | 0.196675 | -0.00516 | 0.000846 | 1E-09 | 8.42E-06 | 3.900398 |
| rs56094641 | hypertension | 16 | 53806453 | G | A | 0.404625 | 0.004028 | 0.000678 | 2.9E-09 | 7.82E-06 | 3.618924 |
| rs16948048 | hypertension | 17 | 47440466 | G | A | 0.366921 | 0.004295 | 0.000691 | 5.1E-10 | 8.57E-06 | 3.96888 |
| rs4291 | hypertension | 17 | 61554194 | A | T | 0.623299 | -0.00392 | 0.000691 | 1.4E-08 | 7.22E-06 | 3.342274 |
| rs62089932 | hypertension | 18 | 72724362 | T | C | 0.859954 | -0.0058 | 0.001027 | 1.6E-08 | 8.1E-06 | 3.750409 |
| rs68096471 | hypertension | 19 | 5175709 | A | G | 0.269191 | -0.00445 | 0.000752 | 3.3E-09 | 7.79E-06 | 3.605992 |
| rs2003476 | hypertension | 19 | 18806668 | C | T | 0.405754 | -0.00399 | 0.000682 | 4.7E-09 | 7.69E-06 | 3.562832 |
| rs167479 | hypertension | 19 | 11526765 | T | G | 0.472463 | -0.00582 | 0.000667 | 2.6E-18 | 1.69E-05 | 7.810691 |
| rs6031435 | hypertension | 20 | 42797358 | G | A | 0.459301 | 0.004188 | 0.000672 | 4.5E-10 | 8.71E-06 | 4.033792 |
| rs1327235 | hypertension | 20 | 10969030 | G | A | 0.476253 | 0.004291 | 0.000667 | 1.3E-10 | 9.19E-06 | 4.253331 |
| rs8118848 | hypertension | 20 | 62461572 | A | G | 0.237633 | -0.00489 | 0.000783 | 4.1E-10 | 8.67E-06 | 4.016297 |
| rs6108171 | hypertension | 20 | 8635551 | T | A | 0.247296 | -0.00749 | 0.000775 | 4.2E-22 | 2.09E-05 | 9.677573 |
| rs6026744 | hypertension | 20 | 57742388 | T | A | 0.119013 | 0.008356 | 0.001032 | 5.7E-16 | 1.46E-05 | 6.778809 |
| rs162395 | hypertension | 21 | 44945759 | C | T | 0.571316 | 0.003877 | 0.000673 | 8.1E-09 | 7.36E-06 | 3.409844 |

Note, Chr, chromosome; EAF, Effect allele frequency; Pos, position; SE, standard error; SNP, single-nucleotide polymorphism. The threshold was set at P < 5×10^-8^

Supplementary Table 7 Characteristics of selected SNPs for overweight according to psychological stress.

| SNP | Trait | Chr | Pos. | Effect allele | Other allele | EAF | Beta | SE | *P* value | R^2^ | *F* statistic |
| --- | --- | --- | --- | --- | --- | --- | --- | --- | --- | --- | --- |
| rs633715 | Overweight | 1 | 1.78E+08 | C | T | 0.267 | 0.078 | 0.011 | 4.00E-12 | 0.002 | 379.197 |
| rs2568958 | Overweight | 1 | 72765116 | A | G | 0.65 | 0.062 | 0.0092 | 1.10E-11 | 0.002 | 278.324 |
| rs12623218 | Overweight | 2 | 632146 | A | T | 0.881 | 0.11 | 0.012 | 5.80E-22 | 0.003 | 404.052 |
| rs10182181 | Overweight | 2 | 25150296 | G | A | 0.5 | 0.057 | 0.009 | 2.10E-10 | 0.002 | 258.477 |
| rs9816226 | Overweight | 3 | 1.86E+08 | T | A | 0.851 | 0.07 | 0.012 | 2.00E-09 | 0.001 | 197.641 |
| rs13130484 | Overweight | 4 | 45175691 | T | C | 0.424 | 0.071 | 0.0094 | 3.90E-14 | 0.002 | 392.104 |
| rs2206277 | Overweight | 6 | 50798526 | T | C | 0.096 | 0.08 | 0.012 | 5.60E-12 | 0.001 | 176.656 |
| rs2596125 | Overweight | 8 | 76642325 | T | C | 0.442 | -0.052 | 0.009 | 5.90E-09 | 0.001 | 212.162 |
| rs2030323 | Overweight | 11 | 27728539 | C | A | 0.783 | 0.079 | 0.011 | 1.10E-12 | 0.002 | 337.616 |
| rs8028313 | Overweight | 15 | 68043057 | G | C | 0.22 | -0.065 | 0.011 | 2.00E-09 | 0.001 | 230.675 |
| rs12444979 | Overweight | 16 | 19933600 | T | C | 0.06 | -0.079 | 0.013 | 1.80E-09 | 0.001 | 111.909 |
| rs1421085 | Overweight | 16 | 53800954 | C | T | 0.448 | 0.14 | 0.0092 | 5.80E-50 | 0.010 | 1554.996 |
| rs523288 | Overweight | 18 | 57848369 | T | A | 0.288 | 0.099 | 0.011 | 1.70E-20 | 0.004 | 641.088 |
| rs10853932 | Overweight | 19 | 34324709 | C | T | 0.691 | 0.067 | 0.011 | 1.30E-09 | 0.002 | 305.102 |

Note, Chr, chromosome; EAF, Effect allele frequency; Pos, position; SE, standard error; SNP, single-nucleotide polymorphism. The threshold was set at P < 5×10^-8^

Supplementary Table 8 Characteristics of selected SNPs for obesity according to psychological stress.

| SNP | Trait | Chr | Pos. | Effect allele | Other allele | EAF | Beta | SE | P value | R^2^ | F statistic |
| --- | --- | --- | --- | --- | --- | --- | --- | --- | --- | --- | --- |
| rs34563765 | Obesity | 1 | 2.26E+08 | C | A | 0.157579 | -0.01467 | 0.000286 | 2.90E-07 | 5.71E-07 | 0.264488 |
| rs2815775 | Obesity | 1 | 72853409 | T | C | 0.799483 | 0.001234 | 0.00026 | 2.10E-06 | 4.88E-07 | 0.225976 |
| rs7565001 | Obesity | 2 | 58802507 | C | T | 0.434196 | -0.001 | 0.00021 | 2.10E-06 | 4.88E-07 | 0.225786 |
| rs834869 | Obesity | 3 | 82665629 | T | C | 0.403898 | 0.000984 | 0.000212 | 3.40E-06 | 4.66E-07 | 0.215672 |
| rs6458093 | Obesity | 6 | 39041825 | G | A | 0.532814 | 0.000991 | 0.000208 | 2.00E-06 | 4.89E-07 | 0.226383 |
| rs7763832 | Obesity | 6 | 34693960 | G | C | 0.779499 | -0.00115 | 0.000251 | 4.70E-06 | 4.54E-07 | 0.21026 |
| rs1243182 | Obesity | 10 | 21916728 | T | C | 0.312017 | 0.001049 | 0.000225 | 3.10E-06 | 4.72E-07 | 0.218751 |
| rs35983068 | Obesity | 11 | 30215430 | C | T | 0.119383 | 0.001695 | 0.000321 | 1.20E-07 | 6.04E-07 | 0.279542 |
| rs11642015 | Obesity | 16 | 53802494 | T | C | 0.403654 | 0.001883 | 0.000212 | 6.80E-19 | 1.71E-06 | 0.7906 |
| rs2726032 | Obesity | 16 | 28338043 | C | T | 0.422049 | 0.001009 | 0.000211 | 1.70E-06 | 4.96E-07 | 0.229747 |
| rs7231987 | Obesity | 18 | 57739840 | T | G | 0.267232 | 0.001495 | 0.000236 | 2.20E-10 | 8.76E-07 | 0.405465 |
| rs10425697 | Obesity | 19 | 54189967 | A | G | 0.18103 | 0.00134 | 0.000273 | 8.90E-07 | 5.33E-07 | 0.246616 |
| rs73137724 | Obesity | 20 | 62308517 | C | T | 0.119303 | 0.001526 | 0.000332 | 4.30E-06 | 4.89E-07 | 0.226634 |

Note, Chr, chromosome; EAF, Effect allele frequency; Pos, position; SE, standard error; SNP, single-nucleotide polymorphism. The threshold was set at P < 5×10^-6^

Supplementary Table 9 Characteristics of selected SNPs for BMI according to psychological stress.

| SNP | Trait | Chr | Pos. | Effect allele | Other allele | EAF | Beta | SE | *P* value | R^2^ | *F* statistic |
| --- | --- | --- | --- | --- | --- | --- | --- | --- | --- | --- | --- |
| rs3127553 | BMI | 1 | 49438005 | A | G | 0.6333 | -0.0259 | 0.0042 | 4.58E-10 | 0.000312 | 53.59825 |
| rs7531118 | BMI | 1 | 72837239 | C | T | 0.6083 | 0.0341 | 0.0041 | 3.9E-17 | 0.000554 | 95.34901 |
| rs17024393 | BMI | 1 | 1.1E+08 | C | T | 0.04167 | 0.0713 | 0.0114 | 3.53E-10 | 0.000406 | 69.85369 |
| rs543874 | BMI | 1 | 1.78E+08 | G | A | 0.2667 | 0.0603 | 0.005 | 9.61E-34 | 0.001422 | 244.9361 |
| rs17381664 | BMI | 1 | 78048331 | C | T | 0.425 | 0.0239 | 0.0041 | 5.31E-09 | 0.000279 | 48.0252 |
| rs11165643 | BMI | 1 | 96924097 | T | C | 0.575 | 0.023 | 0.004 | 5.76E-09 | 0.000259 | 44.47542 |
| rs1016287 | BMI | 2 | 59305625 | C | T | 0.675 | -0.0254 | 0.0044 | 5.87E-09 | 0.000283 | 48.69371 |
| rs6548237 | BMI | 2 | 621461 | C | A | 0.8667 | 0.0687 | 0.0052 | 3.55E-40 | 0.001091 | 187.7507 |
| rs10182181 | BMI | 2 | 25150296 | G | A | 0.5 | 0.0366 | 0.0039 | 3.91E-21 | 0.00067 | 115.2626 |
| rs13098327 | BMI | 3 | 85820181 | A | G | 0.1833 | 0.0333 | 0.0049 | 1.12E-11 | 0.000332 | 57.11537 |
| rs1516725 | BMI | 3 | 1.86E+08 | C | T | 0.9083 | 0.0466 | 0.0059 | 1.91E-15 | 0.000362 | 62.23331 |
| rs16851483 | BMI | 3 | 1.41E+08 | T | G | 0.0917 | 0.0524 | 0.0096 | 4.81E-08 | 0.000457 | 78.69647 |
| rs10938397 | BMI | 4 | 45182527 | G | A | 0.4333 | 0.0404 | 0.0041 | 2.98E-23 | 0.000802 | 137.9584 |
| rs2112347 | BMI | 5 | 75015242 | G | T | 0.375 | -0.0298 | 0.0041 | 3.15E-13 | 0.000416 | 71.61763 |
| rs12529728 | BMI | 6 | 50896630 | G | A | 0.1 | 0.0462 | 0.0051 | 1.76E-19 | 0.000384 | 66.09805 |
| rs9462027 | BMI | 6 | 34797241 | A | G | 0.2583 | 0.0273 | 0.0044 | 4.6E-10 | 0.000286 | 49.12441 |
| rs6465468 | BMI | 7 | 95169514 | T | G | 0.325 | 0.0245 | 0.0045 | 4.98E-08 | 0.000263 | 45.30321 |
| rs2060604 | BMI | 8 | 76650334 | C | T | 0.4417 | -0.0232 | 0.004 | 4.06E-09 | 0.000265 | 45.66481 |
| rs10733682 | BMI | 9 | 1.29E+08 | G | A | 0.575 | -0.0229 | 0.0041 | 1.66E-08 | 0.000256 | 44.08942 |
| rs10968576 | BMI | 9 | 28414339 | G | A | 0.2917 | 0.0289 | 0.0043 | 1.03E-11 | 0.000345 | 59.37376 |
| rs1928295 | BMI | 9 | 1.2E+08 | C | T | 0.425 | -0.0258 | 0.0039 | 3.42E-11 | 0.000325 | 55.9671 |
| rs10767664 | BMI | 11 | 27725986 | A | T | 0.7833 | 0.0456 | 0.0054 | 1.88E-17 | 0.000706 | 121.4839 |
| rs3817334 | BMI | 11 | 47650993 | T | C | 0.45 | 0.0265 | 0.004 | 2.47E-11 | 0.000348 | 59.80166 |
| rs4929923 | BMI | 11 | 8639200 | C | T | 0.7167 | 0.0235 | 0.0041 | 9.05E-09 | 0.000224 | 38.57558 |
| rs7138803 | BMI | 12 | 50247468 | A | G | 0.4417 | 0.0348 | 0.0041 | 1.8E-17 | 0.000597 | 102.7799 |
| rs7141420 | BMI | 14 | 79899454 | T | C | 0.6167 | 0.0262 | 0.0039 | 1.45E-11 | 0.000325 | 55.82794 |
| rs4981693 | BMI | 14 | 29680331 | A | G | 0.7917 | 0.0336 | 0.0054 | 4.9E-10 | 0.000372 | 64.05979 |
| rs745213 | BMI | 15 | 68060389 | G | T | 0.8417 | 0.0343 | 0.0053 | 1.31E-10 | 0.000314 | 53.93342 |
| rs9931989 | BMI | 16 | 28906084 | C | G | 0.7083 | -0.0258 | 0.0042 | 9.47E-10 | 0.000275 | 47.31598 |
| rs12446632 | BMI | 16 | 19935389 | A | G | 0.1333 | -0.0432 | 0.0058 | 1.45E-13 | 0.000431 | 74.19063 |
| rs1121980 | BMI | 16 | 53809247 | A | G | 0.475 | 0.0774 | 0.0039 | 4.87E-86 | 0.002988 | 515.3826 |
| rs663129 | BMI | 18 | 57838401 | A | G | 0.2833 | 0.0567 | 0.0046 | 3.49E-34 | 0.001306 | 224.8085 |
| rs8097783 | BMI | 18 | 58051294 | A | G | 0.1167 | -0.0512 | 0.0076 | 2.19E-11 | 0.00054 | 92.99275 |
| rs11663558 | BMI | 18 | 21133937 | A | G | 0.5 | -0.027 | 0.0046 | 4.37E-09 | 0.000365 | 62.70774 |
| rs7239883 | BMI | 18 | 40147671 | A | G | 0.6833 | -0.0231 | 0.0041 | 1.51E-08 | 0.000231 | 39.72639 |
| rs2303108 | BMI | 19 | 47589895 | C | T | 0.675 | 0.0278 | 0.0043 | 8.53E-11 | 0.000339 | 58.33367 |
| rs6091540 | BMI | 20 | 51087862 | T | C | 0.275 | -0.0297 | 0.0044 | 2.15E-11 | 0.000352 | 60.51063 |

Note, BMI, body mass index; Chr, chromosome; EAF, Effect allele frequency; Pos, position; SE, standard error; SNP, single-nucleotide polymorphism. The threshold was set at *P* < 5×10^-8^

Supplementary Table 10 Characteristics of selected SNPs for hyperlipidemia according to psychological stress.

| SNP | Trait | Chr | Pos. | Effect allele | Other allele | EAF | Beta | SE | P value | R^2^ | F statistic |
| --- | --- | --- | --- | --- | --- | --- | --- | --- | --- | --- | --- |
| rs10889335 | Hyperlipidemia | 1 | 62960101 | G | A | 0.355227 | -0.00088 | 0.000187 | 2.30E-06 | 3.58E-07 | 0.16573 |
| rs4970834 | Hyperlipidemia | 1 | 1.1E+08 | T | C | 0.186677 | -0.00119 | 0.00023 | 2.50E-07 | 4.27E-07 | 0.197748 |
| rs12613844 | Hyperlipidemia | 2 | 2.41E+08 | G | T | 0.243534 | -0.00098 | 0.000208 | 2.50E-06 | 3.54E-07 | 0.163823 |
| rs9356756 | Hyperlipidemia | 6 | 20844151 | A | G | 0.593231 | -0.00088 | 0.000182 | 1.30E-06 | 3.76E-07 | 0.17431 |
| rs56393506 | Hyperlipidemia | 6 | 1.61E+08 | T | C | 0.171301 | 0.001502 | 0.000241 | 5.00E-10 | 6.4E-07 | 0.296372 |
| rs6471516 | Hyperlipidemia | 8 | 96100477 | C | G | 0.813109 | -0.00108 | 0.00023 | 2.40E-06 | 3.57E-07 | 0.165308 |
| rs10757277 | Hyperlipidemia | 9 | 22124450 | G | A | 0.482972 | 0.000946 | 0.000178 | 1.10E-07 | 4.47E-07 | 0.207148 |
| rs163607 | Hyperlipidemia | 11 | 72205699 | C | T | 0.516335 | -0.00087 | 0.00018 | 1.50E-06 | 3.77E-07 | 0.174337 |
| rs159696 | Hyperlipidemia | 12 | 29734477 | T | C | 0.677786 | -0.0009 | 0.000192 | 2.70E-06 | 3.53E-07 | 0.163515 |
| rs117073308 | Hyperlipidemia | 13 | 41275319 | C | T | 0.137527 | 0.001212 | 0.000263 | 4.20E-06 | 3.49E-07 | 0.16137 |
| rs12721051 | Hyperlipidemia | 19 | 45422160 | G | C | 0.188348 | 0.001444 | 0.000228 | 2.60E-10 | 6.37E-07 | 0.294982 |

Note, Chr, chromosome; EAF, Effect allele frequency; Pos, position; SE, standard error; SNP, single-nucleotide polymorphism. The threshold was set at P < 5×10^-6^.

Supplementary Table 11 Characteristics of selected SNPs for HDL-C according to psychological stress.

| SNP | Trait | Chr | Pos. | Effect allele | Other allele | EAF | Beta | SE | *P* value | R^2^ | *F* statistic |
| --- | --- | --- | --- | --- | --- | --- | --- | --- | --- | --- | --- |
| rs12748152 | HDL-C | 1 | 26811902 | T | C | 0.09 | 0.051 | 0.006354 | 1E-15 | 0.000426 | 80.37545 |
| rs1689800 | HDL-C | 1 | 1.82E+08 | G | A | 0.35 | 0.034 | 0.00371 | 5E-20 | 0.000526 | 99.23888 |
| rs4650994 | HDL-C | 1 | 1.79E+08 | G | A | 0.49 | 0.021 | 0.003626 | 7E-09 | 0.00022 | 41.57332 |
| rs4846914 | HDL-C | 1 | 2.3E+08 | G | A | 0.41 | 0.048 | 0.003574 | 4E-41 | 0.001115 | 210.4344 |
| rs12145743 | HDL-C | 1 | 1.57E+08 | G | T | 0.34 | 0.02 | 0.003564 | 2E-08 | 0.00018 | 33.85906 |
| rs4660293 | HDL-C | 1 | 39562508 | G | A | 0.24 | 0.035 | 0.004018 | 3E-18 | 0.000447 | 84.30807 |
| rs2972146 | HDL-C | 2 | 2.26E+08 | G | T | 0.37 | 0.032 | 0.003767 | 2E-17 | 0.000477 | 90.06659 |
| rs1047891 | HDL-C | 2 | 2.11E+08 | A | C | 0.33 | 0.027 | 0.004407 | 9E-10 | 0.000322 | 60.80936 |
| rs12328675 | HDL-C | 2 | 1.65E+08 | C | T | 0.13 | 0.045 | 0.005667 | 2E-15 | 0.000458 | 86.41731 |
| rs2290547 | HDL-C | 3 | 47019693 | A | G | 0.2 | 0.03 | 0.005098 | 4E-09 | 0.000288 | 54.32525 |
| rs13326165 | HDL-C | 3 | 52498102 | A | G | 0.21 | 0.029 | 0.004473 | 9E-11 | 0.000279 | 52.63537 |
| rs2013208 | HDL-C | 3 | 50091966 | T | C | 0.5 | 0.025 | 0.003665 | 9E-12 | 0.000313 | 58.94811 |
| rs17404153 | HDL-C | 3 | 1.32E+08 | T | G | 0.14 | 0.028 | 0.004789 | 5E-09 | 0.000189 | 35.60727 |
| rs2606736 | HDL-C | 3 | 11358775 | C | T | 0.39 | 0.025 | 0.004586 | 5E-08 | 0.000297 | 56.09417 |
| rs6805251 | HDL-C | 3 | 1.2E+08 | T | C | 0.39 | 0.02 | 0.00349 | 1E-08 | 0.00019 | 35.89643 |
| rs13107325 | HDL-C | 4 | 1.02E+08 | T | C | 0.08 | 0.071 | 0.008845 | 1E-15 | 0.000742 | 140.0332 |
| rs2602836 | HDL-C | 4 | 99093654 | A | G | 0.44 | 0.019 | 0.003485 | 5E-08 | 0.000178 | 33.55361 |
| rs10019888 | HDL-C | 4 | 26061368 | G | A | 0.18 | 0.027 | 0.004953 | 5E-08 | 0.000215 | 40.59023 |
| rs3822072 | HDL-C | 4 | 88820118 | A | G | 0.46 | 0.025 | 0.003604 | 4E-12 | 0.000311 | 58.57072 |
| rs6450176 | HDL-C | 5 | 54002195 | A | G | 0.26 | 0.025 | 0.004054 | 7E-10 | 0.000241 | 45.3632 |
| rs1936800 | HDL-C | 6 | 1.27E+08 | C | T | 0.49 | 0.02 | 0.003175 | 3E-10 | 0.0002 | 37.70745 |
| rs998584 | HDL-C | 6 | 43790159 | A | C | 0.49 | 0.026 | 0.003877 | 2E-11 | 0.000338 | 63.73439 |
| rs605066 | HDL-C | 6 | 1.4E+08 | C | T | 0.42 | 0.028 | 0.005053 | 3E-08 | 0.000382 | 72.05654 |
| rs4142995 | HDL-C | 7 | 17879635 | T | G | 0.38 | 0.026 | 0.003811 | 9E-12 | 0.000319 | 60.08616 |
| rs17173637 | HDL-C | 7 | 1.51E+08 | C | T | 0.12 | 0.036 | 0.006415 | 2E-08 | 0.000274 | 51.62998 |
| rs17145738 | HDL-C | 7 | 73568544 | T | C | 0.13 | 0.041 | 0.005675 | 5E-13 | 0.00038 | 71.73145 |
| rs702485 | HDL-C | 7 | 6409641 | G | A | 0.45 | 0.024 | 0.003489 | 6E-12 | 0.000285 | 53.78184 |
| rs4917014 | HDL-C | 7 | 50266267 | G | T | 0.32 | 0.022 | 0.003839 | 1E-08 | 0.000211 | 39.7292 |
| rs4731702 | HDL-C | 7 | 1.31E+08 | T | C | 0.49 | 0.029 | 0.003458 | 5E-17 | 0.00042 | 79.2974 |
| rs9987289 | HDL-C | 8 | 9325848 | A | G | 0.1 | 0.082 | 0.006082 | 2E-41 | 0.00121 | 228.5127 |
| rs12678919 | HDL-C | 8 | 19986711 | G | A | 0.13 | 0.155 | 0.005948 | 1E-149 | 0.005434 | 1030.402 |
| rs581080 | HDL-C | 9 | 15305380 | G | C | 0.21 | 0.042 | 0.004621 | 1E-19 | 0.000585 | 110.4367 |
| rs1883025 | HDL-C | 9 | 1.05E+08 | T | C | 0.25 | 0.07 | 0.004098 | 2E-65 | 0.001838 | 347.1444 |
| rs970548 | HDL-C | 10 | 45517829 | C | A | 0.26 | 0.026 | 0.004087 | 2E-10 | 0.00026 | 49.0658 |
| rs7941030 | HDL-C | 11 | 1.23E+08 | C | T | 0.39 | 0.027 | 0.003489 | 1E-14 | 0.000347 | 65.43148 |
| rs2923084 | HDL-C | 11 | 10367235 | G | A | 0.18 | 0.026 | 0.004769 | 5E-08 | 0.0002 | 37.63863 |
| rs12801636 | HDL-C | 11 | 65623846 | A | G | 0.23 | 0.024 | 0.004331 | 3E-08 | 0.000204 | 38.48077 |
| rs499974 | HDL-C | 11 | 75743976 | A | C | 0.19 | 0.026 | 0.004537 | 1E-08 | 0.000208 | 39.24549 |
| rs3136441 | HDL-C | 11 | 46721697 | C | T | 0.18 | 0.054 | 0.004842 | 7E-29 | 0.000861 | 162.4658 |
| rs11246602 | HDL-C | 11 | 54607190 | C | T | 0.15 | 0.034 | 0.005345 | 2E-10 | 0.000295 | 55.60453 |
| rs174546 | HDL-C | 11 | 61802358 | T | C | 0.36 | 0.039 | 0.003567 | 8E-28 | 0.000701 | 132.2605 |
| rs964184 | HDL-C | 11 | 1.17E+08 | C | G | 0.84 | 0.106 | 0.007286 | 6E-48 | 0.00302 | 571.2665 |
| rs7134375 | HDL-C | 12 | 20320824 | A | C | 0.43 | 0.021 | 0.003664 | 1E-08 | 0.000216 | 40.77462 |
| rs4759375 | HDL-C | 12 | 1.23E+08 | T | C | 0.08 | 0.056 | 0.010106 | 3E-08 | 0.000462 | 87.09004 |
| rs838880 | HDL-C | 12 | 1.25E+08 | C | T | 0.34 | 0.048 | 0.00408 | 6E-32 | 0.001034 | 195.195 |
| rs4765127 | HDL-C | 12 | 1.24E+08 | T | G | 0.35 | 0.032 | 0.005208 | 8E-10 | 0.000466 | 87.90182 |
| rs11613352 | HDL-C | 12 | 57398797 | T | C | 0.26 | 0.028 | 0.00381 | 2E-13 | 0.000302 | 56.90708 |
| rs7134594 | HDL-C | 12 | 1.1E+08 | C | T | 0.48 | 0.035 | 0.004763 | 2E-13 | 0.000612 | 115.3879 |
| rs4983559 | HDL-C | 14 | 1.05E+08 | G | A | 0.4 | 0.02 | 0.00349 | 1E-08 | 0.000192 | 36.21335 |
| rs2652834 | HDL-C | 15 | 63104668 | A | G | 0.21 | 0.028 | 0.00424 | 4E-11 | 0.00026 | 49.067 |
| rs1532085 | HDL-C | 15 | 58391167 | A | G | 0.4 | 0.107 | 0.003652 | 1E-188 | 0.005496 | 1042.044 |
| rs3764261 | HDL-C | 16 | 56959412 | A | C | 0.32 | 0.241 | 0.004788 | 5.00E-090 | 0.025277 | 4890.191 |
| rs1121980 | HDL-C | 16 | 53775335 | A | G | 0.43 | 0.02 | 0.003454 | 7E-09 | 0.000196 | 36.98304 |
| rs16942887 | HDL-C | 16 | 67894139 | A | G | 0.14 | 0.083 | 0.005373 | 8E-54 | 0.001659 | 313.3414 |
| rs2925979 | HDL-C | 16 | 81501185 | T | C | 0.31 | 0.035 | 0.003851 | 1E-19 | 0.000524 | 98.87549 |
| rs11869286 | HDL-C | 17 | 39657603 | G | C | 0.35 | 0.032 | 0.003789 | 3E-17 | 0.000466 | 87.90182 |
| rs4148008 | HDL-C | 17 | 68879153 | G | C | 0.33 | 0.028 | 0.003927 | 1E-12 | 0.000347 | 65.39876 |
| rs4129767 | HDL-C | 17 | 78407903 | G | A | 0.48 | 0.024 | 0.003579 | 2E-11 | 0.000288 | 54.2383 |
| rs12967135 | HDL-C | 18 | 60181790 | A | G | 0.25 | 0.026 | 0.004735 | 4E-08 | 0.000254 | 47.81588 |
| rs7241918 | HDL-C | 18 | 49634583 | G | T | 0.19 | 0.09 | 0.006414 | 1E-44 | 0.002493 | 471.3265 |
| rs731839 | HDL-C | 19 | 33408159 | G | A | 0.35 | 0.022 | 0.003709 | 3E-09 | 0.00022 | 41.53713 |
| rs386000 | HDL-C | 19 | 54288907 | C | G | 0.26 | 0.048 | 0.004833 | 3E-23 | 0.000887 | 167.335 |
| rs7255436 | HDL-C | 19 | 8368312 | C | A | 0.47 | 0.032 | 0.005702 | 2E-08 | 0.00051 | 96.25192 |
| rs4420638 | HDL-C | 19 | 44919689 | G | A | 0.19 | 0.067 | 0.007049 | 2E-21 | 0.001382 | 260.9173 |
| rs17695224 | HDL-C | 19 | 51820963 | A | G | 0.26 | 0.029 | 0.003946 | 2E-13 | 0.000324 | 61.04579 |
| rs737337 | HDL-C | 19 | 11236817 | C | T | 0.11 | 0.056 | 0.006677 | 5E-17 | 0.000614 | 115.8616 |
| rs6065906 | HDL-C | 20 | 45925376 | C | T | 0.19 | 0.059 | 0.004455 | 5E-40 | 0.001071 | 202.2657 |
| rs1800961 | HDL-C | 20 | 44413724 | T | C | 0.05 | 0.127 | 0.010379 | 2E-34 | 0.001532 | 289.3884 |
| rs181362 | HDL-C | 22 | 21577779 | T | C | 0.23 | 0.038 | 0.004378 | 4E-18 | 0.000511 | 96.49883 |

Note, HDL-C, high density lipoprotein cholesterol; Chr, chromosome; EAF, Effect allele frequency; Pos, position; SE, standard error; SNP, single-nucleotide polymorphism. The threshold was set at P < 5×10^-8^

Supplementary Table 12 Characteristics of selected SNPs for TG according to psychological stress.

| SNP | Trait | Chr | Pos. | Effect allele | Other allele | EAF | Beta | SE | *P* value | R^2^ | *F* statistic |
| --- | --- | --- | --- | --- | --- | --- | --- | --- | --- | --- | --- |
| rs4846914 | TG | 1 | 2.3E+08 | G | A | 0.41 | 0.04 | 0.003462 | 7.00E-31 | 0.000774 | 146.0852 |
| rs12748152 | TG | 1 | 26811902 | T | C | 0.09 | 0.037 | 0.006056 | 1E-09 | 0.000224 | 42.29596 |
| rs2131925 | TG | 1 | 62560271 | G | T | 0.34 | 0.066 | 0.00362 | 3.00E-74 | 0.001955 | 369.3811 |
| rs1260326 | TG | 2 | 27508073 | T | C | 0.39 | 0.115 | 0.00348 | 2.00E-239 | 0.006292 | 1194.114 |
| rs2972146 | TG | 2 | 2.26E+08 | G | T | 0.37 | 0.028 | 0.003548 | 3.00E-15 | 0.000366 | 68.94951 |
| rs645040 | TG | 3 | 1.36E+08 | G | T | 0.23 | 0.029 | 0.004123 | 2.00E-12 | 0.000298 | 56.18987 |
| rs442177 | TG | 4 | 87109109 | G | T | 0.42 | 0.031 | 0.003509 | 1.00E-18 | 0.000468 | 88.33202 |
| rs6831256 | TG | 4 | 3471412 | G | A | 0.42 | 0.026 | 0.003696 | 2.00E-12 | 0.000329 | 62.12711 |
| rs9686661 | TG | 5 | 56565959 | T | C | 0.2 | 0.038 | 0.004649 | 3.00E-16 | 0.000462 | 87.17702 |
| rs6882076 | TG | 5 | 1.57E+08 | T | C | 0.36 | 0.029 | 0.003652 | 2.00E-15 | 0.000388 | 73.10733 |
| rs1936800 | TG | 6 | 1.27E+08 | C | T | 0.49 | 0.02 | 0.003609 | 3E-08 | 0.0002 | 37.70745 |
| rs998584 | TG | 6 | 43790159 | A | C | 0.49 | 0.49 | 0.062097 | 3.00E-15 | 0.120002 | 25715.25 |
| rs4722551 | TG | 7 | 25952206 | C | T | 0.2 | 0.023 | 0.003548 | 9.00E-11 | 0.000169 | 31.92738 |
| rs17145738 | TG | 7 | 73568544 | T | C | 0.13 | 0.115 | 0.005452 | 9.00E-99 | 0.002991 | 565.8138 |
| rs13238203 | TG | 7 | 72664689 | T | C | 0.04 | 0.059 | 0.012632 | 3E-08 | 0.000267 | 50.42727 |
| rs38855 | TG | 7 | 1.17E+08 | G | A | 0.47 | 0.019 | 0.003386 | 2E-08 | 0.00018 | 33.92135 |
| rs1495741 | TG | 8 | 18415371 | G | A | 0.26 | 0.04 | 0.005733 | 3.00E-12 | 0.000616 | 116.1734 |
| rs11776767 | TG | 8 | 10826419 | C | G | 0.37 | 0.022 | 0.00331 | 3.00E-11 | 0.000226 | 42.55982 |
| rs12678919 | TG | 8 | 19986711 | G | A | 0.13 | 0.17 | 0.005642 | 2.00E-199 | 0.006537 | 1240.86 |
| rs2068888 | TG | 10 | 93079885 | A | G | 0.45 | 0.024 | 0.003579 | 2.00E-11 | 0.000285 | 53.78184 |
| rs1832007 | TG | 10 | 5212884 | G | A | 0.18 | 0.033 | 0.004691 | 2.00E-12 | 0.000321 | 60.64123 |
| rs174546 | TG | 11 | 61802358 | T | C | 0.36 | 0.045 | 0.003498 | 7.00E-38 | 0.000933 | 176.1275 |
| rs964184 | TG | 11 | 1.17E+08 | C | G | 0.84 | 0.234 | 0.007326 | 7.00E-224 | 0.014718 | 2816.986 |
| rs11613352 | TG | 12 | 57398797 | T | C | 0.26 | 0.028 | 0.003756 | 9.00E-14 | 0.000302 | 56.90708 |
| rs4765127 | TG | 12 | 1.24E+08 | T | G | 0.35 | 0.029 | 0.005167 | 2E-08 | 0.000383 | 72.18679 |
| rs2929282 | TG | 15 | 43953733 | T | A | 0.07 | 0.072 | 0.012004 | 2E-09 | 0.000675 | 127.3659 |
| rs1532085 | TG | 15 | 58391167 | A | G | 0.4 | 0.031 | 0.00354 | 2.00E-18 | 0.000461 | 87.02602 |
| rs2412710 | TG | 15 | 42391589 | A | G | 0.04 | 0.099 | 0.014763 | 2.00E-11 | 0.000753 | 142.0505 |
| rs3764261 | TG | 16 | 56959412 | A | C | 0.32 | 0.04 | 0.003839 | 2.00E-25 | 0.000696 | 131.4 |
| rs3198697 | TG | 16 | 15036083 | T | C | 0.43 | 0.02 | 0.003564 | 2E-08 | 0.000196 | 36.98304 |
| rs1121980 | TG | 16 | 53775335 | A | G | 0.43 | 0.021 | 0.00379 | 3E-08 | 0.000216 | 40.77462 |
| rs8077889 | TG | 17 | 43800798 | C | A | 0.22 | 0.025 | 0.004362 | 1E-08 | 0.000215 | 40.45802 |
| rs10401969 | TG | 19 | 19296909 | C | T | 0.09 | 0.121 | 0.006855 | 1.00E-69 | 0.002398 | 453.3269 |
| rs7248104 | TG | 19 | 7224420 | A | G | 0.42 | 0.022 | 0.003537 | 5.00E-10 | 0.000236 | 44.47738 |
| rs731839 | TG | 19 | 33408159 | G | A | 0.35 | 0.022 | 0.003709 | 3E-09 | 0.00022 | 41.53713 |
| rs6065906 | TG | 20 | 45925376 | C | T | 0.19 | 0.053 | 0.004332 | 2.00E-34 | 0.000865 | 163.185 |
| rs5756931 | TG | 22 | 38150026 | C | T | 0.4 | 0.02 | 0.003609 | 3E-08 | 0.000192 | 36.21335 |

Note, TG, triglycerides; Chr, chromosome; EAF, Effect allele frequency; Pos, position; SE, standard error; SNP, single-nucleotide polymorphism. The threshold was set at P < 5×10^-8^

Supplementary Table 13 Characteristics of selected SNPs for FBG according to psychological stress.

| SNP | Trait | Chr | Pos. | Effect allele | Other allele | EAF | Beta | SE | *P* value | R^2^ | *F* statistic |
| --- | --- | --- | --- | --- | --- | --- | --- | --- | --- | --- | --- |
| rs2075423 | FBG | 1 | 2.14E+08 | T | G | 0.3679 | 0.0161 | 0.001701 | 3.00E-21 | 0.000121 | 33.9309 |
| rs348330 | FBG | 1 | 2.30E+08 | A | G | 0.6276 | 0.0122 | 0.001937 | 3.00E-10 | 6.96E-05 | 19.58025 |
| rs6662924 | FBG | 1 | 1.00E+08 | A | C | 0.187 | 0.0143 | 0.00227 | 3.00E-10 | 6.22E-05 | 17.49875 |
| rs78132593 | FBG | 1 | 1.51E+08 | A | C | 0.2084 | 0.0147 | 0.002334 | 3.00E-10 | 7.13E-05 | 20.06526 |
| rs841572 | FBG | 1 | 42970380 | A | G | 0.4081 | 0.0089 | 0.001771 | 5.00E-09 | 3.83E-05 | 10.76929 |
| rs115640879 | FBG | 2 | 28045875 | T | C | 0.0766 | 0.0241 | 0.003541 | 1.00E-11 | 8.22E-05 | 23.12406 |
| rs1260326 | FBG | 2 | 27508073 | T | C | 0.3872 | 0.0282 | 0.001655 | 4.00E-65 | 0.000377 | 106.2409 |
| rs1371614 | FBG | 2 | 26930006 | T | C | 0.2511 | 0.0158 | 0.001953 | 6.00E-16 | 9.39E-05 | 26.42416 |
| rs1402837 | FBG | 2 | 1.69E+08 | T | C | 0.3974 | 0.0502 | 0.004167 | 2.00E-33 | 0.001207 | 340.0671 |
| rs145353824 | FBG | 2 | 1.69E+08 | A | C | 0.9748 | 0.0766 | 0.011423 | 2.00E-11 | 0.000288 | 81.14738 |
| rs189548 | FBG | 2 | 54713975 | A | G | 0.7302 | 0.0123 | 0.002074 | 3.00E-09 | 5.96E-05 | 16.77628 |
| rs540524 | FBG | 2 | 1.69E+08 | A | G | 0.4301 | 0.0388 | 0.002617 | 1.00E-49 | 0.000738 | 207.8394 |
| rs6731931 | FBG | 2 | 1.73E+08 | T | C | 0.7955 | 0.012 | 0.002308 | 2.00E-08 | 4.69E-05 | 13.18536 |
| rs7582529 | FBG | 2 | 1.69E+08 | T | G | 0.1864 | 0.0531 | 0.007396 | 7.00E-13 | 0.000855 | 240.8758 |
| rs11708067 | FBG | 3 | 1.23E+08 | A | G | 0.7717 | 0.0281 | 0.002034 | 2.00E-43 | 0.000278 | 78.31836 |
| rs13064576 | FBG | 3 | 49604997 | T | C | 0.3183 | 0.0119 | 0.002196 | 6.00E-09 | 6.15E-05 | 17.29523 |
| rs1604038 | FBG | 3 | 1.71E+08 | T | C | 0.2912 | 0.0198 | 0.001801 | 4.00E-28 | 0.000162 | 45.55032 |
| rs16851397 | FBG | 3 | 1.41E+08 | A | G | 0.953 | 0.0327 | 0.004586 | 1.00E-12 | 9.58E-05 | 26.95899 |
| rs17437560 | FBG | 3 | 1.52E+08 | T | C | 0.1056 | 0.0175 | 0.003158 | 3.00E-08 | 5.78E-05 | 16.28068 |
| rs189651013 | FBG | 3 | 1.93E+08 | A | T | 0.0074 | 0.11 | 0.021157 | 2.00E-08 | 0.000178 | 50.03158 |
| rs4132537 | FBG | 3 | 1.01E+08 | A | G | 0.5208 | 0.0093 | 0.001725 | 7.00E-10 | 4.32E-05 | 12.14921 |
| rs6808574 | FBG | 3 | 1.88E+08 | T | C | 0.3907 | 0.0127 | 0.001696 | 7.00E-14 | 7.68E-05 | 21.61181 |
| rs4862423 | FBG | 4 | 1.85E+08 | T | C | 0.3964 | 0.0123 | 0.001967 | 4.00E-10 | 7.24E-05 | 20.37512 |
| rs157512 | FBG | 5 | 56513300 | T | C | 0.7588 | 0.0134 | 0.002155 | 5.00E-10 | 6.57E-05 | 18.49773 |
| rs1820176 | FBG | 5 | 96360881 | T | C | 0.7081 | 0.0247 | 0.002019 | 2.00E-34 | 0.000252 | 70.99174 |
| rs3733977 | FBG | 5 | 1.69E+08 | A | G | 0.1615 | 0.0101 | 0.002231 | 6.00E-09 | 2.76E-05 | 7.775101 |
| rs7708285 | FBG | 5 | 77130042 | A | G | 0.7252 | 0.0133 | 0.002177 | 1.00E-09 | 7.05E-05 | 19.84194 |
| rs7729395 | FBG | 5 | 1.03E+08 | T | C | 0.0493 | 0.0232 | 0.004187 | 3.00E-08 | 5.05E-05 | 14.1992 |
| rs10305492 | FBG | 6 | 39079018 | A | G | 0.0144 | 0.0763 | 0.009473 | 8.00E-16 | 0.000165 | 46.51143 |
| rs12055786 | FBG | 6 | 1.53E+08 | T | C | 0.4242 | 0.012 | 0.001763 | 1.00E-11 | 7.03E-05 | 19.79753 |
| rs1761880 | FBG | 6 | 1.17E+08 | A | C | 0.6765 | 0.0095 | 0.001915 | 7.00E-09 | 3.95E-05 | 11.11686 |
| rs2025704 | FBG | 6 | 43848640 | T | C | 0.5535 | 0.0083 | 0.001746 | 2.00E-08 | 3.41E-05 | 9.582653 |
| rs3778321 | FBG | 6 | 7250037 | A | G | 0.1915 | 0.0186 | 0.002202 | 3.00E-17 | 0.000107 | 30.15067 |
| rs9348441 | FBG | 6 | 20680447 | A | T | 0.2827 | 0.0176 | 0.001916 | 4.00E-20 | 0.000126 | 35.35756 |
| rs1635852 | FBG | 7 | 28149792 | T | C | 0.5052 | 0.0082 | 0.001539 | 1.00E-08 | 3.36E-05 | 9.460433 |
| rs2108349 | FBG | 7 | 50718966 | A | G | 0.5242 | 0.0252 | 0.004971 | 4.00E-08 | 0.000317 | 89.1735 |
| rs2908286 | FBG | 7 | 44195138 | T | C | 0.1766 | 0.0626 | 0.008239 | 3.00E-14 | 0.00114 | 321.0858 |
| rs2971670 | FBG | 7 | 44186502 | T | C | 0.198 | 0.0574 | 0.004947 | 4.00E-31 | 0.001046 | 294.7771 |
| rs58925536 | FBG | 7 | 76025256 | T | C | 0.0339 | 0.0306 | 0.005261 | 6.00E-09 | 6.13E-05 | 17.26104 |
| rs878521 | FBG | 7 | 44216044 | A | G | 0.2444 | 0.0549 | 0.001951 | 3.00E-174 | 0.001113 | 313.6153 |
| rs12541643 | FBG | 8 | 80164639 | T | C | 0.4763 | 0.0118 | 0.002018 | 5.00E-09 | 6.95E-05 | 19.54938 |
| rs13266634 | FBG | 8 | 1.17E+08 | T | C | 0.4303 | 0.0313 | 0.003859 | 5.00E-16 | 0.00048 | 135.2355 |
| rs3757970 | FBG | 8 | 1.44E+08 | A | G | 0.6263 | 0.0111 | 0.002208 | 5.00E-09 | 5.77E-05 | 16.23126 |
| rs7012637 | FBG | 8 | 9315699 | A | G | 0.4738 | 0.018 | 0.001753 | 1.00E-24 | 0.000162 | 45.47124 |
| rs896854 | FBG | 8 | 94948283 | T | C | 0.5012 | 0.0099 | 0.001702 | 6.00E-09 | 4.90E-05 | 13.79129 |
| rs9650069 | FBG | 8 | 1.17E+08 | T | C | 0.3206 | 0.0286 | 0.001784 | 8.00E-58 | 0.000356 | 100.3117 |
| rs9987289 | FBG | 8 | 9325848 | A | G | 0.2016 | 0.0359 | 0.00674 | 1.00E-08 | 0.000415 | 116.8035 |
| rs10811660 | FBG | 9 | 22134069 | A | G | 0.1761 | 0.0223 | 0.002168 | 8.00E-25 | 0.000144 | 40.61458 |
| rs10974438 | FBG | 9 | 4291928 | A | C | 0.6398 | 0.0198 | 0.001718 | 1.00E-30 | 0.000181 | 50.85955 |
| rs3829109 | FBG | 9 | 1.36E+08 | A | G | 0.3019 | 0.0163 | 0.002031 | 1.00E-15 | 0.000112 | 31.51957 |
| rs507666 | FBG | 9 | 1.33E+08 | A | G | 0.1956 | 0.0164 | 0.001965 | 7.00E-17 | 8.46E-05 | 23.81996 |
| rs642157 | FBG | 9 | 33344254 | A | T | 0.8526 | 0.0132 | 0.002459 | 8.00E-09 | 4.38E-05 | 12.32496 |
| rs1046521 | FBG | 10 | 63154707 | A | G | 0.6965 | 0.01 | 0.001871 | 9.00E-09 | 4.23E-05 | 11.898 |
| rs12784552 | FBG | 10 | 1.11E+08 | A | G | 0.9098 | 0.0329 | 0.00283 | 3.00E-31 | 0.000178 | 50.00312 |
| rs2839671 | FBG | 10 | 26216893 | A | G | 0.1716 | 0.016 | 0.002142 | 8.00E-14 | 7.28E-05 | 20.48352 |
| rs34872471 | FBG | 10 | 1.13E+08 | T | C | 0.658 | 0.0332 | 0.007273 | 0.000005 | 0.000496 | 139.6752 |
| rs61875120 | FBG | 10 | 1.13E+08 | T | C | 0.7703 | 0.0765 | 0.013349 | 1.00E-08 | 0.002071 | 584.0094 |
| rs7095788 | FBG | 10 | 93624395 | T | C | 0.3538 | 0.0106 | 0.001767 | 2.00E-09 | 5.14E-05 | 14.45888 |
| rs7903146 | FBG | 10 | 1.13E+08 | T | C | 0.2717 | 0.0259 | 0.002085 | 2.00E-35 | 0.000265 | 74.72923 |
| rs10501320 | FBG | 11 | 47272248 | C | G | 0.2518 | 0.0219 | 0.001934 | 1.00E-29 | 0.000181 | 50.86462 |
| rs10743026 | FBG | 11 | 7554514 | C | G | 0.7612 | 0.0109 | 0.002228 | 1.00E-08 | 4.32E-05 | 12.15572 |
| rs10769572 | FBG | 11 | 49291683 | A | G | 0.2529 | 0.0105 | 0.001912 | 4.00E-08 | 4.17E-05 | 11.72465 |
| rs10838524 | FBG | 11 | 45848626 | A | G | 0.4542 | 0.0238 | 0.001788 | 2.00E-40 | 0.000281 | 79.05553 |
| rs1483121 | FBG | 11 | 48311808 | A | G | 0.1429 | 0.0159 | 0.002588 | 8.00E-10 | 6.19E-05 | 17.42852 |
| rs174583 | FBG | 11 | 61842278 | T | C | 0.3583 | 0.0168 | 0.001732 | 3.00E-22 | 0.00013 | 36.5283 |
| rs2168101 | FBG | 11 | 8233861 | A | C | 0.3199 | 0.0125 | 0.002256 | 3.00E-08 | 6.80E-05 | 19.13429 |
| rs2237896 | FBG | 11 | 2837210 | A | G | 0.3698 | 0.0251 | 0.004013 | 4.00E-10 | 0.000294 | 82.66011 |
| rs3842753 | FBG | 11 | 2159830 | T | G | 0.2806 | 0.0134 | 0.002259 | 3.00E-09 | 7.25E-05 | 20.4021 |
| rs61909476 | FBG | 11 | 1.28E+08 | A | C | 0.0689 | 0.0812 | 0.014788 | 4.00E-08 | 0.000846 | 238.2708 |
| rs7115753 | FBG | 11 | 45890462 | A | G | 0.7318 | 0.0271 | 0.004395 | 7.00E-10 | 0.000288 | 81.1504 |
| rs77464186 | FBG | 11 | 72749353 | A | C | 0.8155 | 0.0231 | 0.002225 | 3.00E-25 | 0.000161 | 45.19493 |
| rs8914 | FBG | 11 | 46677574 | A | G | 0.107 | 0.0195 | 0.002688 | 4.00E-13 | 7.27E-05 | 20.45087 |
| rs11610045 | FBG | 12 | 1.32E+08 | A | G | 0.5009 | 0.0144 | 0.001974 | 3.00E-13 | 0.000104 | 29.17993 |
| rs12315434 | FBG | 12 | 57387153 | A | C | 0.7712 | 0.011 | 0.002003 | 4.00E-08 | 4.27E-05 | 12.01718 |
| rs12315677 | FBG | 12 | 18524304 | T | C | 0.8399 | 0.0387 | 0.007048 | 4.00E-08 | 0.000403 | 113.3944 |
| rs2657879 | FBG | 12 | 56471554 | A | G | 0.8199 | 0.0119 | 0.002055 | 7.00E-09 | 4.18E-05 | 11.76962 |
| rs6489811 | FBG | 12 | 1.21E+08 | A | G | 0.4665 | 0.011 | 0.001854 | 3.00E-09 | 6.02E-05 | 16.95014 |
| rs6538804 | FBG | 12 | 97455132 | C | G | 0.6086 | 0.0142 | 0.001905 | 9.00E-14 | 9.61E-05 | 27.03628 |
| rs11619319 | FBG | 13 | 27913462 | A | G | 0.7789 | 0.0173 | 0.001877 | 3.00E-20 | 0.000103 | 29.01236 |
| rs576674 | FBG | 13 | 32980164 | A | G | 0.8447 | 0.0179 | 0.00251 | 1.00E-12 | 8.41E-05 | 23.65877 |
| rs12888855 | FBG | 14 | 1.00E+08 | A | C | 0.213 | 0.0135 | 0.001962 | 6.00E-12 | 6.11E-05 | 17.19587 |
| rs35889227 | FBG | 14 | 89589124 | T | G | 0.6184 | 0.013 | 0.002064 | 3.00E-10 | 7.98E-05 | 22.44786 |
| rs12594062 | FBG | 15 | 74810510 | T | C | 0.3595 | 0.0098 | 0.001676 | 5.00E-09 | 4.42E-05 | 12.44701 |
| rs6598541 | FBG | 15 | 98727906 | A | G | 0.3635 | 0.0114 | 0.001643 | 4.00E-12 | 6.01E-05 | 16.92444 |
| rs7163757 | FBG | 15 | 62099409 | T | C | 0.4511 | 0.0217 | 0.001726 | 3.00E-36 | 0.000233 | 65.63908 |
| rs7178572 | FBG | 15 | 77454848 | A | G | 0.2994 | 0.0121 | 0.001962 | 7.00E-10 | 6.14E-05 | 17.28602 |
| rs9940149 | FBG | 16 | 250642 | A | G | 0.1722 | 0.0123 | 0.002309 | 1.00E-08 | 4.31E-05 | 12.13845 |
| rs12452315 | FBG | 17 | 47402080 | A | C | 0.5252 | 0.0095 | 0.001827 | 2.00E-08 | 4.50E-05 | 12.66712 |
| rs1880900 | FBG | 17 | 17440808 | T | C | 0.4244 | 0.0107 | 0.002291 | 3.00E-08 | 5.59E-05 | 15.74214 |
| rs3764400 | FBG | 17 | 48046570 | T | C | 0.8405 | 0.0116 | 0.00244 | 2.00E-08 | 3.61E-05 | 10.15327 |
| rs1337918 | FBG | 20 | 22586970 | A | C | 0.158 | 0.0509 | 0.006191 | 2.00E-16 | 0.000689 | 194.1243 |
| rs17265513 | FBG | 20 | 41203988 | T | C | 0.7908 | 0.0158 | 0.002098 | 5.00E-14 | 8.26E-05 | 23.24631 |
| rs6113722 | FBG | 20 | 22576461 | A | G | 0.0449 | 0.0424 | 0.004121 | 8.00E-25 | 0.000154 | 43.39798 |
| rs39713 | FBG | 22 | 29947197 | T | C | 0.0871 | 0.0169 | 0.003011 | 2.00E-08 | 4.54E-05 | 12.78233 |

Note, FBG, fasting blood glucose; Chr, chromosome; EAF, Effect allele frequency; Pos, position; SE, standard error; SNP, single-nucleotide polymorphism. The threshold was set at *P* < 5×10^-8^

Supplementary Table 14 Reverse causal relationships of MetS and its components with psychological stress performed using MR

| Exposure | Outcome | nSNPs | Method | OR (95%CI) | *P* | Q pval | Intercept pval | Global *P* |
| --- | --- | --- | --- | --- | --- | --- | --- | --- |
| MetS | Psychological stress | 68 | IVW | 1.041 (0.979, 1.107) | 0.196 | 1.197e-06 |  |  |
|  |  | 68 | MR Egger | 0.975 (0.872, 1.089) | 0.654 |  | 0.169 |  |
|  |  | 68 | MR-PRESSO | 1.035 (0.977, 1.095) | 0.244 |  |  | < 0.001 |
|  |  | 68 | Weighted median | 0.987 (0.917, 1.062) | 0.722 |  |  |  |
|  |  | 68 | Simple mode | 1.076 (0.910, 1.271) | 0.395 |  |  |  |
|  |  | 68 | Weighted mode | 0.988 (0.921, 1.059) | 0.730 |  |  |  |
| Hypertension | Psychological stress | 66 | IVW | 2.386 (1.209, 4.710) | 0.012 | 0.021 |  |  |
|  |  | 66 | MR Egger | 7.946 (0.655, 96.432) | 0.109 |  | 0.330 |  |
|  |  | 66 | MR-PRESSO | 2.388 (1.241, 4.598) | 0.011 |  |  | 0.051 |
|  |  | 66 | Weighted median | 2.822 (1.148, 6.941) | 0.024 |  |  |  |
|  |  | 66 | Simple mode | 12.028 (1.265, 114.415) | 0.034 |  |  |  |
|  |  | 66 | Weighted mode | 10.666 (1.349, 84.359) | 0.028 |  |  |  |
| Overweight | Psychological stress | 14 | IVW | 0.972 (0.873, 1.083) | 0.611 | 0.064 |  |  |
|  |  | 14 | MR Egger | 0.767 (0.541, 1.087) | 0.161 |  | 0.187 |  |
|  |  | 14 | MR-PRESSO | 0.943 (0.866, 1.026) | 0.199 |  |  | 0.650 |
|  |  | 14 | Weighted median | 0.986 (0.863, 1.127) | 0.841 |  |  |  |
|  |  | 14 | Simple mode | 1.030 (0.830, 1.278) | 0.796 |  |  |  |
|  |  | 14 | Weighted mode | 0.996 (0.854, 1.162) | 0.960 |  |  |  |
| Obesity | Psychological stress | 13 | IVW | 0.910 (0.002, 512.00) | 0.977 | 0.187 |  |  |
|  |  | 13 | MR Egger | 5.02E-05 (6.04E-17, 4.17E+07) | 0.494 |  | 0.486 |  |
|  |  | 13 | MR-PRESSO | 0.910 (0.002, 512.284) | 0.977 |  |  | 0.219 |
|  |  | 13 | Weighted median | 0.215 (8.15E-05, 5.68E+02) | 0.702 |  |  |  |
|  |  | 13 | Simple mode | 0.036 (5.00E-07, 2.63E+03) | 0.572 |  |  |  |
|  |  | 13 | Weighted mode | 0.111 (5.60E-06, 2.21E+03) | 0.671 |  |  |  |
| BMI | Psychological stress | 37 | IVW | 0.959 (0.842, 1.092) | 0.526 | 0.097 |  |  |
|  |  | 37 | MR Egger | 0.879 (0.618, 1.251) | 0.479 |  | 0.607 |  |
|  |  | 37 | MR-PRESSO | 0.959 (0.842, 1.092) | 0.530 |  |  | 0.107 |
|  |  | 37 | Weighted median | 0.959 (0.794, 1.157) | 0.662 |  |  |  |
|  |  | 37 | Simple mode | 1.086 (0.753, 1.567) | 0.661 |  |  |  |
|  |  | 37 | Weighted mode | 1.005 (0.769, 1.315) | 0.969 |  |  |  |
| Hyperlipidaemia | Psychological stress | 11 | IVW | 9.257 (0.008, 1.10E+04) | 0.538 | 0.464 |  |  |
|  |  | 11 | MR Egger | 3.41E+08 (8.85E-08, 1.31E+24) | 0.311 |  | 0.357 |  |
|  |  | 11 | MR-PRESSO | 9.257 (0.009, 1.00E+04) | 0.546 |  |  | 0.472 |
|  |  | 11 | Weighted median | 28.053 (0.001, 6.02E+05) | 0.512 |  |  |  |
|  |  | 11 | Simple mode | 4.06E+08 (1.09E-04, 1.51E+09) | 0.454 |  |  |  |
|  |  | 11 | Weighted mode | 3.42E+02 (3.09E-04, 3.78E+08) | 0.430 |  |  |  |
| HDL-C | Psychological stress | 69 | IVW | 1.049 (0.966, 1.139) | 0.259 | 0.066 |  |  |
|  |  | 69 | MR Egger | 0.998 (0.849, 1.173) | 0.981 |  | 0.485 |  |
|  |  | 69 | MR-PRESSO | 1.038 (0.955, 1.128) | 0.389 |  |  | 0.051 |
|  |  | 69 | Weighted median | 1.011 (0.901, 1.135) | 0.852 |  |  |  |
|  |  | 69 | Simple mode | 0.985 (0.799, 1.213) | 0.884 |  |  |  |
|  |  | 69 | Weighted mode | 0.996 (0.877, 1.131) | 0.954 |  |  |  |
| TG | Psychological stress | 31 | IVW | 1.015 (0.970, 1.061) | 0.523 | 0.100 |  |  |
|  |  | 31 | MR Egger | 1.012 (0.959, 1.068) | 0.663 |  | 0.857 |  |
|  |  | 31 | MR-PRESSO | 1.010 (0.965, 1.056) | 0.678 |  |  | 0.171 |
|  |  | 31 | Weighted median | 1.010 (0.967, 1.054) | 0.668 |  |  |  |
|  |  | 31 | Simple mode | 0.939 (0.786, 1.123) | 0.542 |  |  |  |
|  |  | 31 | Weighted mode | 1.008 (0.966, 1.051) | 0.716 |  |  |  |
| FBG | Psychological stress | 94 | IVW | 1.001 (0.875, 1.145) | 0.988 | 0.020 |  |  |
|  |  | 94 | MR Egger | 0.919 (0.123, 1.168) | 0.492 |  | 0.400 |  |
|  |  | 94 | MR-PRESSO | 1.003 (0.888, 1.113) | 0.965 |  |  | 0.018 |
|  |  | 94 | Weighted median | 1.045 (0.867, 1.260) | 0.643 |  |  |  |
|  |  | 94 | Simple mode | 1.210 (0.858, 1.706) | 0.279 |  |  |  |
|  |  | 94 | Weighted mode | 1.022 (0.858, 1.216) | 0.811 |  |  |  |

Note: MetS, metabolic syndrome; MR: Mendelian randomization; nSNPs: number of single-nucleotide polymorphisms; OR: odds ratio; CI: confidence interval; Q pval: P value of the Cochran Q statistic; IVW: inverse-variance weighted; MR-PRESSO: MR pleiotropy residual sum and outlier; BMI: body mass index; FBG: fasting blood-glucose; HDL-C: high-density lipoprotein cholesterol; TG, triglycerides.


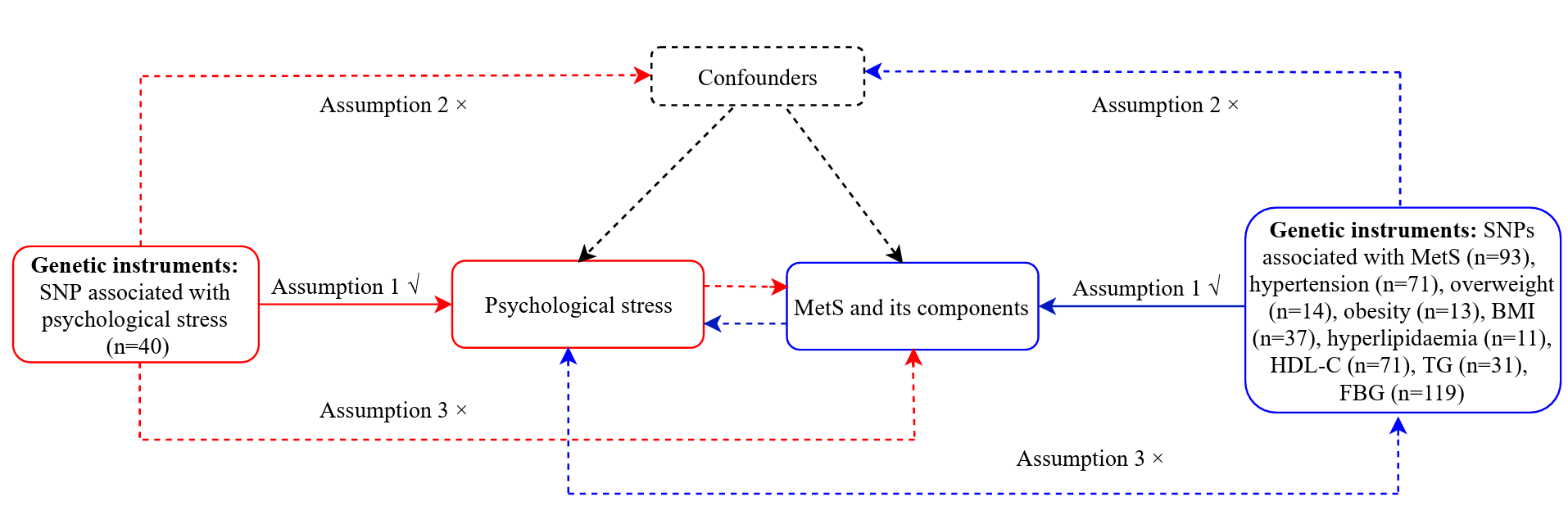


Supplementary Figure 1 Diagram of the hypothesis of instrumental variables in a Mendelian randomization study. SNP, single nucleotide polymorphism; MetS, metabolic syndrome; BMI, body mass index; FBG: fasting blood-glucose; HDL-C, high-density lipoprotein cholesterol; TG, triglycerides.


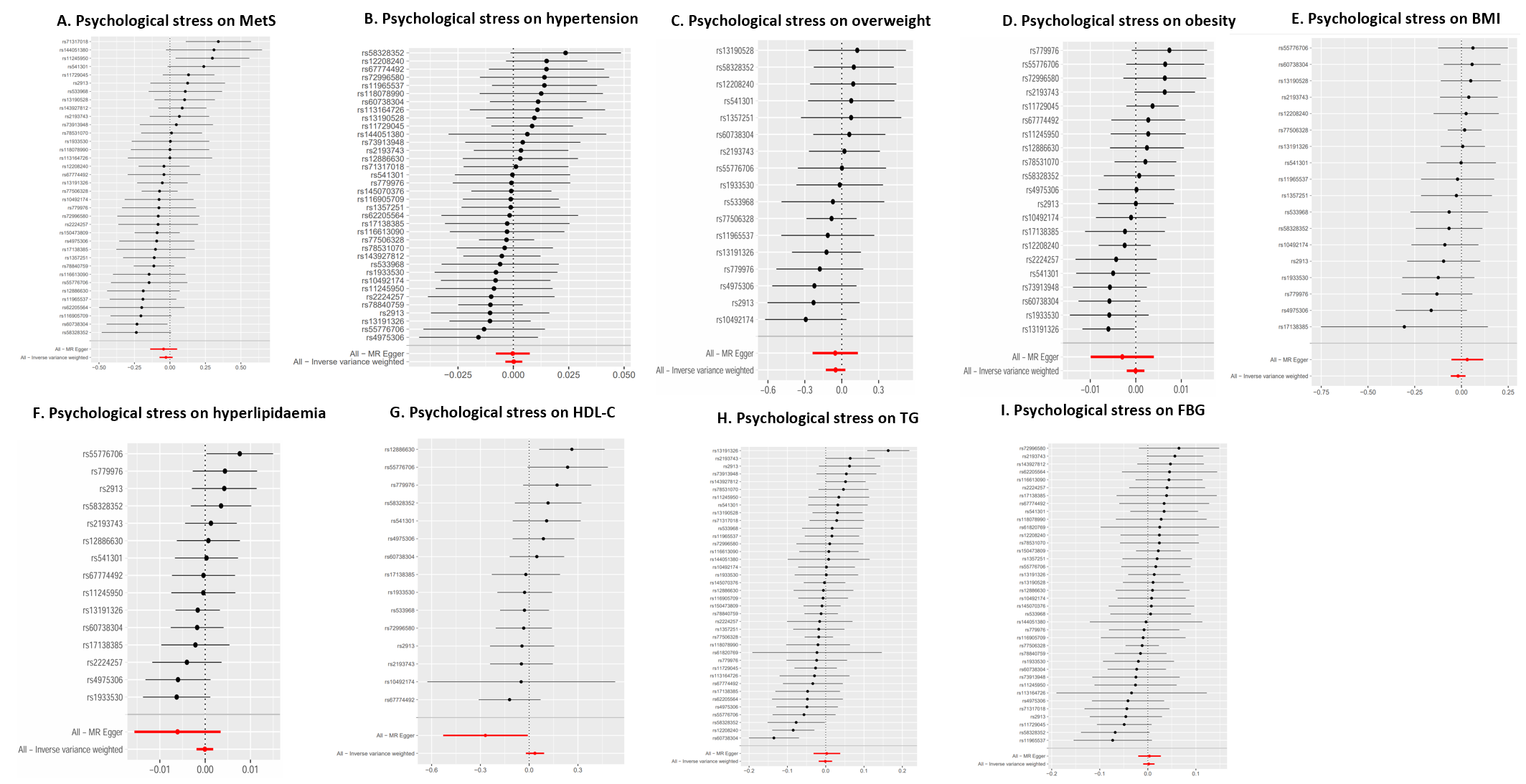


Supplementary Figure 2 The forest plots of the association between genetically predicted psychological stress on MetS and its components in MR analysis. MR, Mendelian randomization; IVW, inverse-variance weighted; MetS, metabolic syndrome; BMI, body mass index; FBG, fasting blood-glucose; HDL-C, high-density lipoprotein cholesterol; TG, triglycerides.


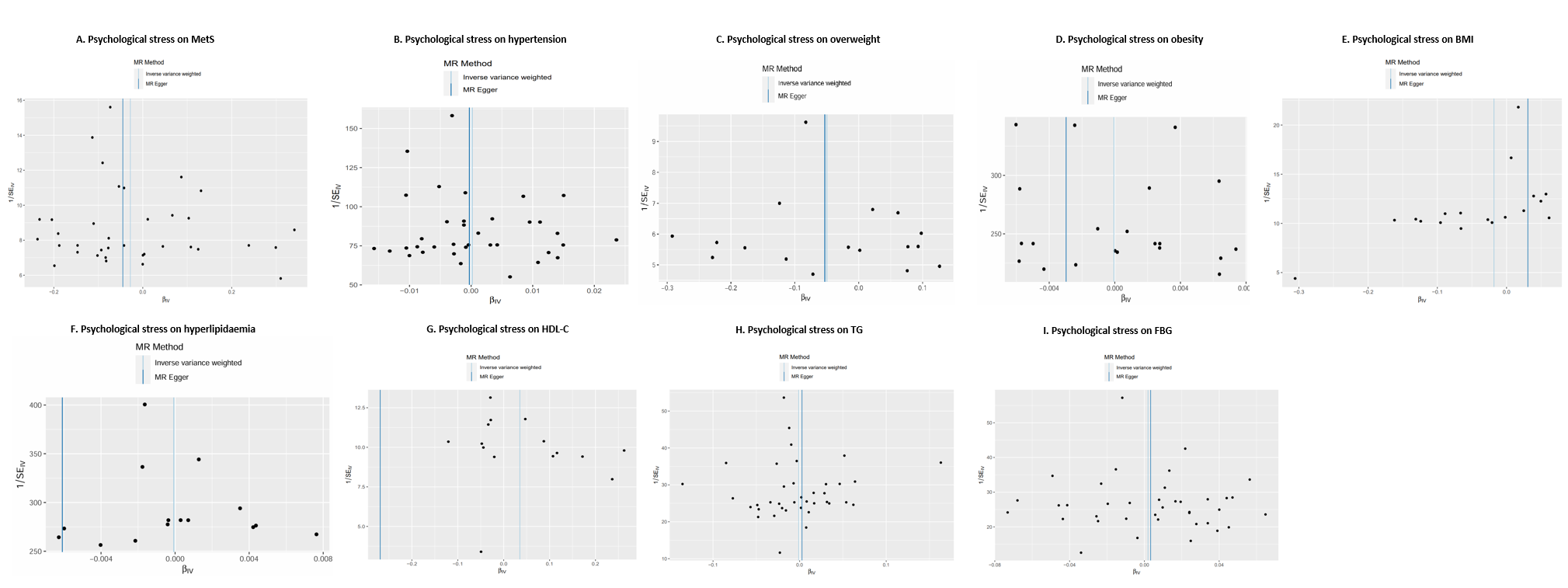


Supplementary Figure 3 The funnel plots of the association between genetically predicted psychological stress on MetS and its components in MR analysis. MR, Mendelian randomization; IVW, inverse-variance weighted; MetS, metabolic syndrome; BMI, body mass index; FBG, fasting blood-glucose; HDL-C, high-density lipoprotein cholesterol; TG, triglycerides.


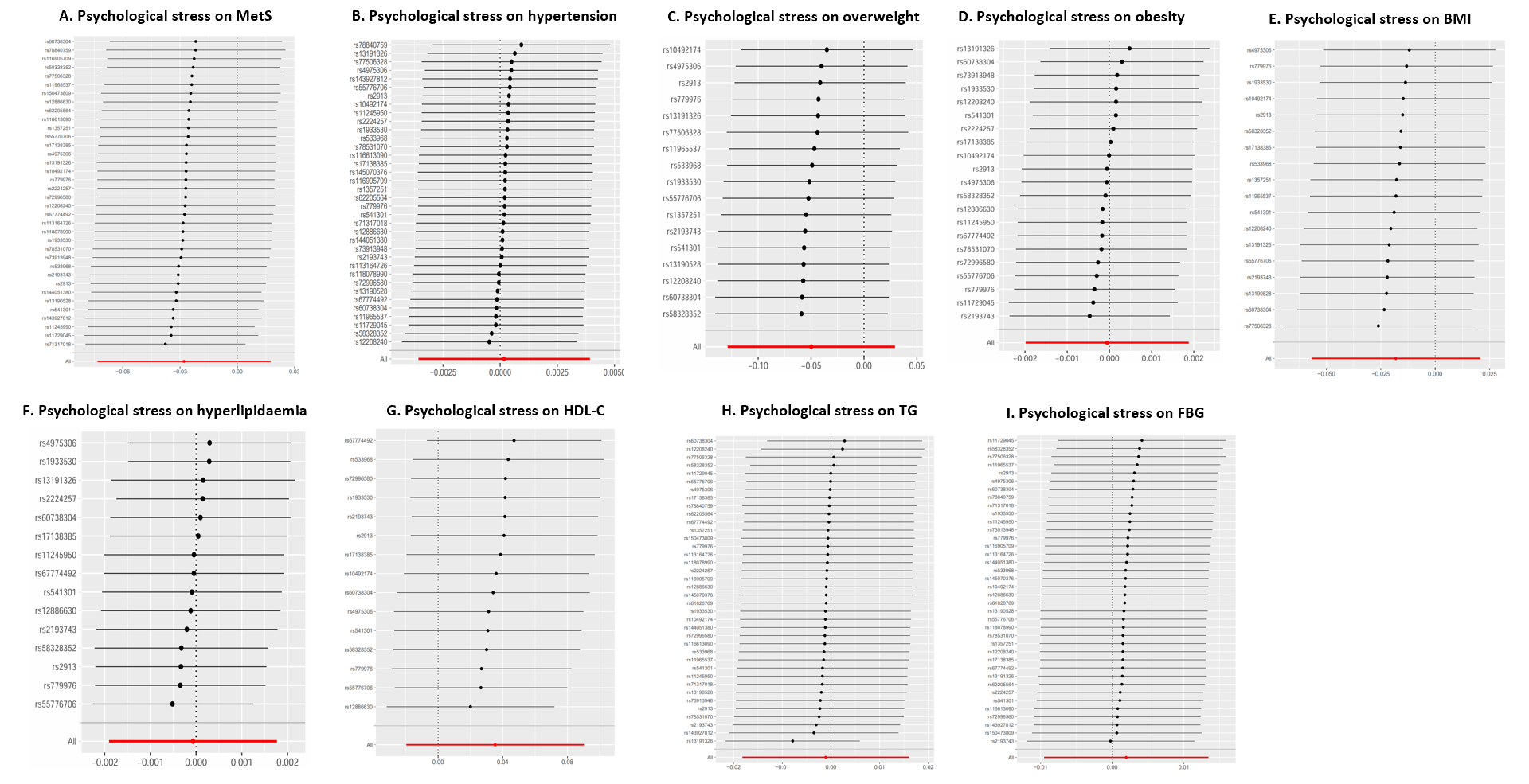


Supplementary Figure 4 The leave-one-out analysis of the association between genetically predicted psychological stress on MetS and its components in MR analysis. MR, Mendelian randomization; MetS, metabolic syndrome; BMI, body mass index; FBG, fasting blood-glucose; HDL-C, high-density lipoprotein cholesterol; TG, triglycerides.


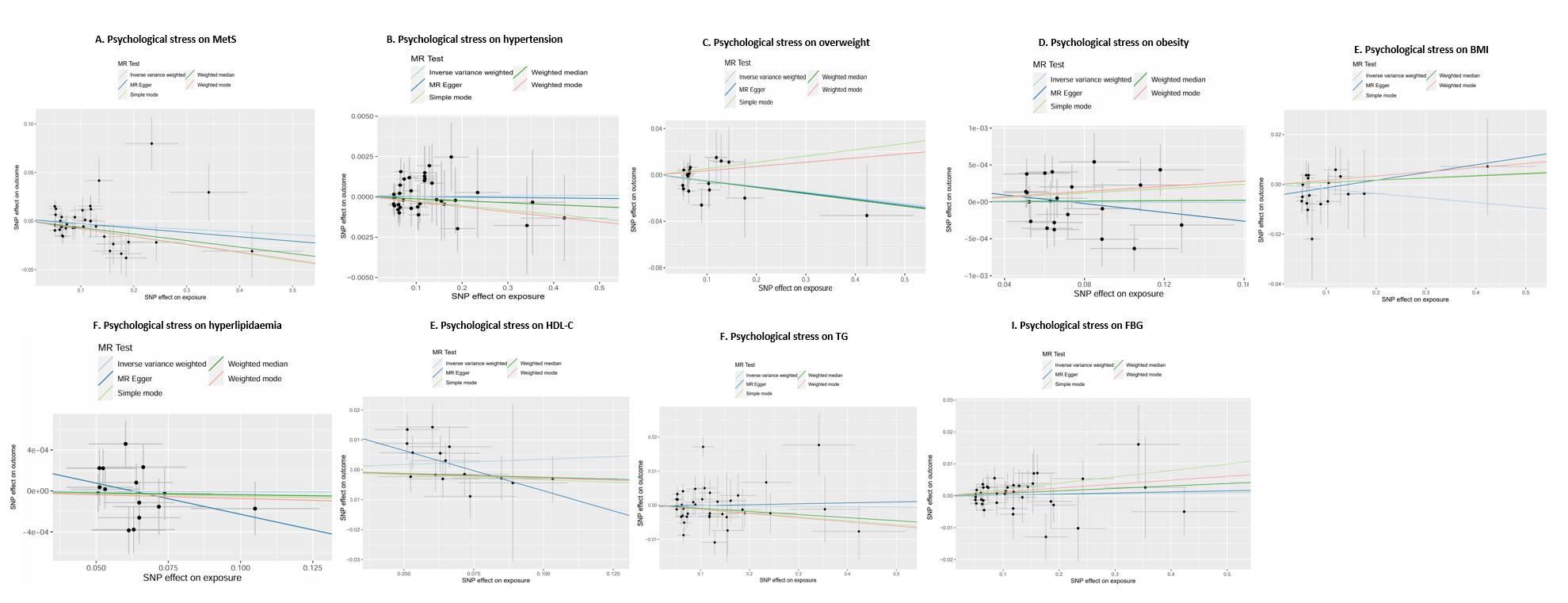


Supplementary Figure 5 The scatter plots of the association between genetically predicted psychological stress on MetS and its components in MR analysis. MR, Mendelian randomization; IVW, inverse-variance weighted; MetS, metabolic syndrome; BMI, body mass index; FBG, fasting blood-glucose; HDL-C, high-density lipoprotein cholesterol; TG, triglycerides.


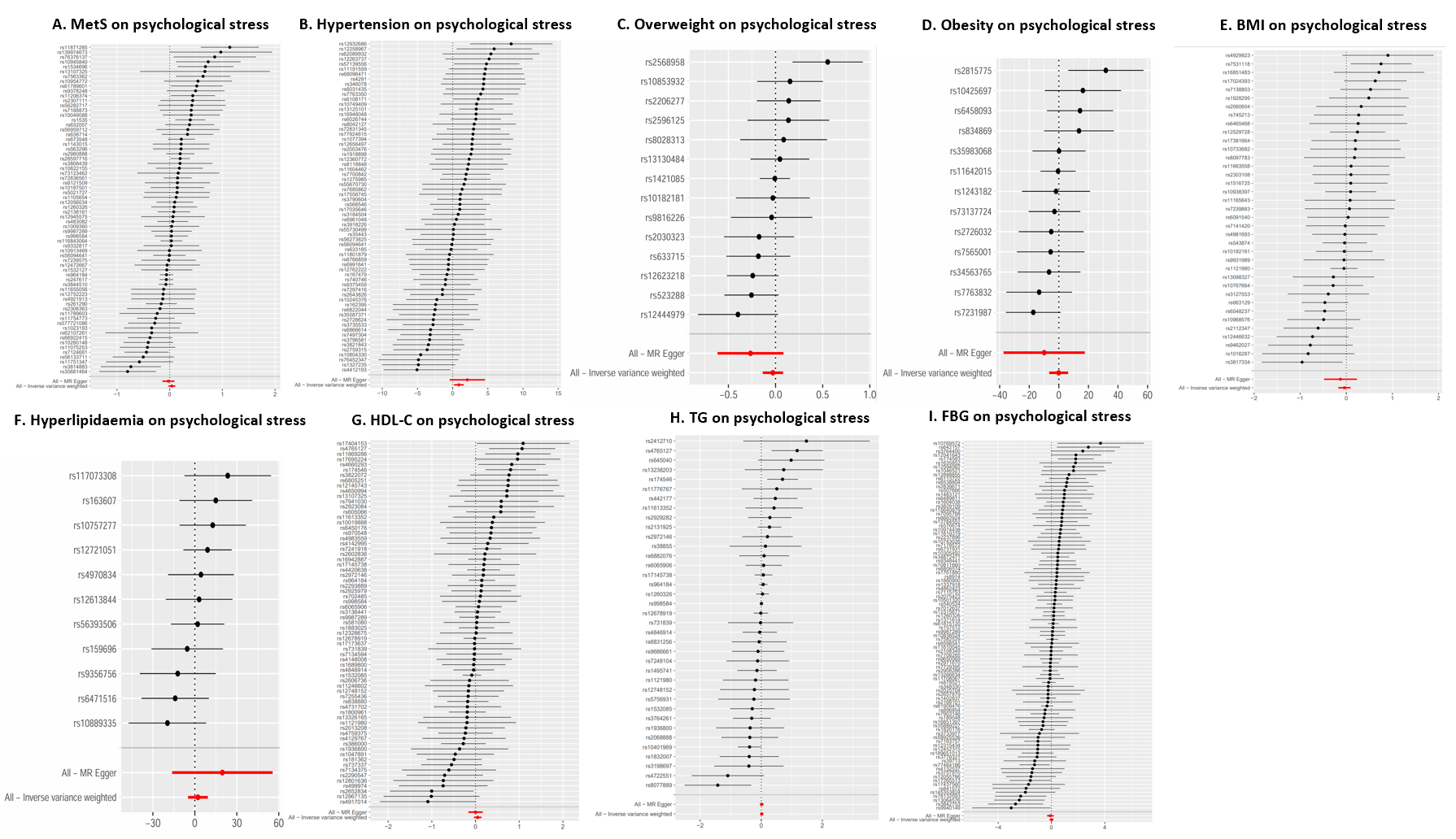


Supplementary Figure 6 The forest plots of the association between genetic predicted MetS and its components on psychological stress in MR analysis. MR, Mendelian randomization; IVW, inverse-variance weighted; MetS, metabolic syndrome; BMI, body mass index; FBG, fasting blood-glucose; HDL-C, high-density lipoprotein cholesterol; TG, triglycerides.


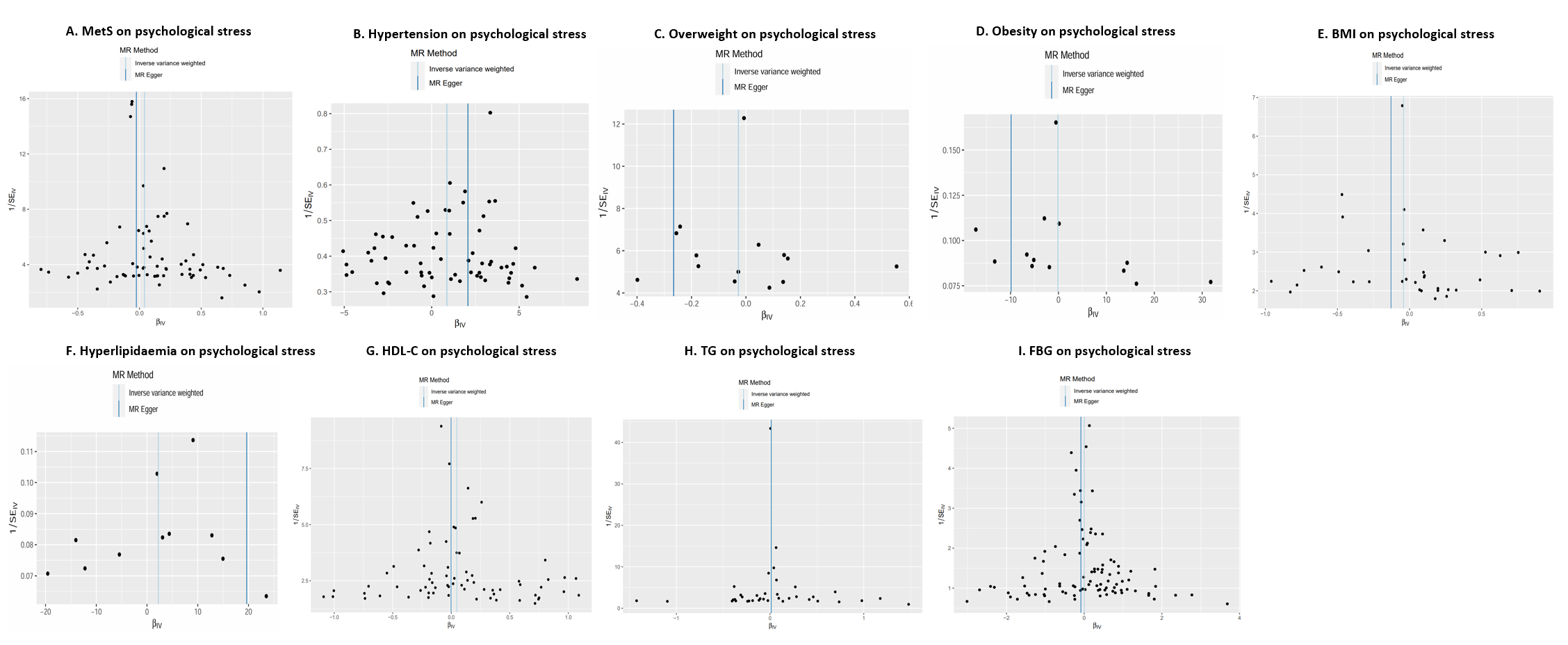


Supplementary Figure 7 The funnel plots of the association between genetically predicted MetS and its components on psychological stress in MR analysis. MR, Mendelian randomization; IVW, inverse-variance weighted; MetS, metabolic syndrome; BMI, body mass index; FBG, fasting blood-glucose; HDL-C, high-density lipoprotein cholesterol; TG, triglycerides.


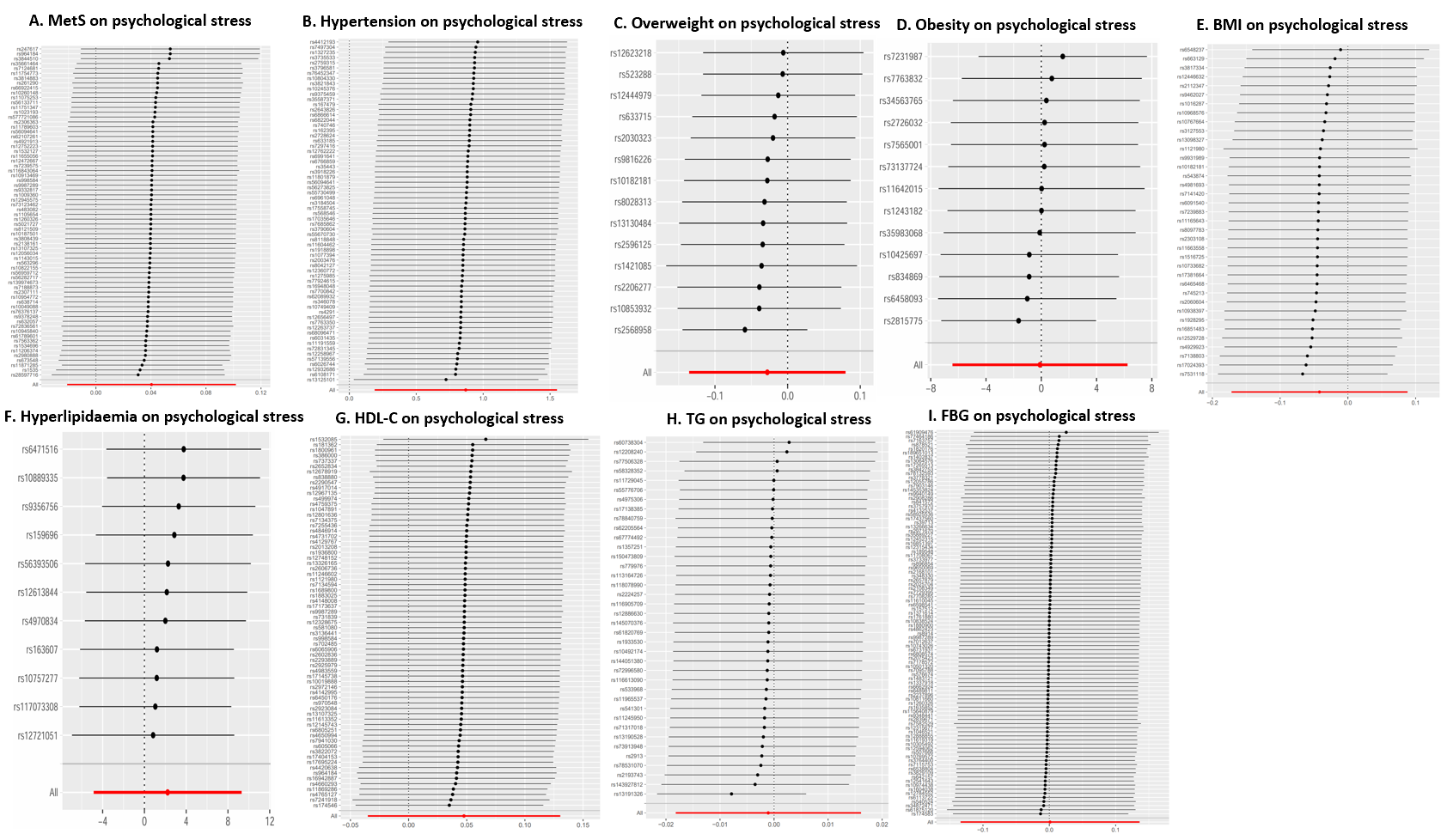


Supplementary Figure 8 The leave-one-out analysis of the association between genetically predicted MetS and its components on psychological stress in MR analysis. MR, Mendelian randomization; MetS, metabolic syndrome; BMI, body mass index; FBG, fasting blood-glucose; HDL-C, high-density lipoprotein cholesterol; TG, triglycerides.


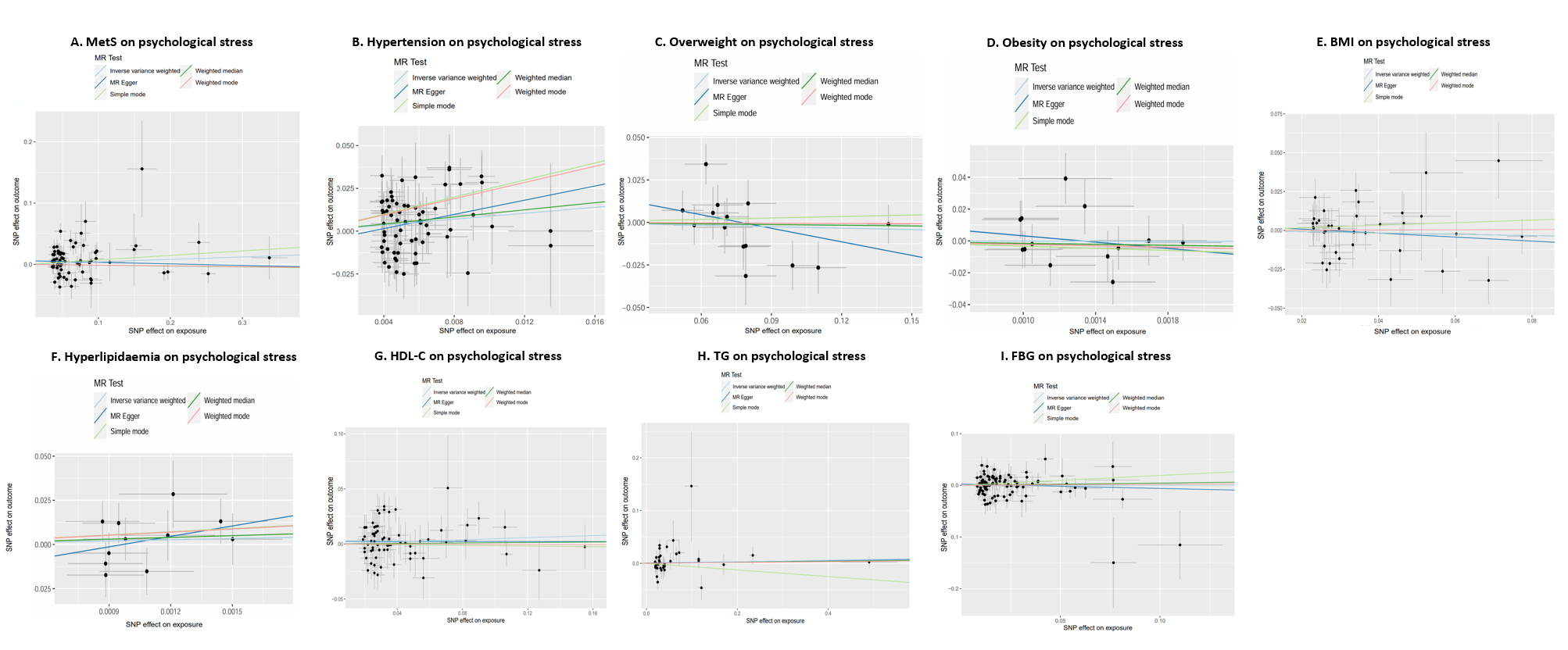


Supplementary Figure 9 The scatter plots of the association between genetically predicted MetS and its components on psychological stress in MR analysis. MR, Mendelian randomization; IVW, inverse-variance weighted; MetS, metabolic syndrome; BMI, body mass index; FBG, fasting blood-glucose; HDL-C, high-density lipoprotein cholesterol; TG, triglycerides.
